# Large-scale integrative network-based analysis identifies common pathways disrupted by copy number alterations across cancers

**DOI:** 10.1186/1471-2164-14-440

**Published:** 2013-07-03

**Authors:** Tae Hyun Hwang, Gowtham Atluri, Rui Kuang, Vipin Kumar, Timothy Starr, Kevin AT Silverstein, Peter M Haverty, Zemin Zhang, Jinfeng Liu

**Affiliations:** 1 Masonic Cancer Center, University of Minnesota – Twin Cities, Minneapolis, MN, USA; 2 Department of Computer Science and Engineering, University of Minnesota – Twin Cities, Minneapolis, MN, USA; 3 Department of Bioinformatics and Computational Biology, Genentech Inc, South San Francisco, CA, USA; 4 Department of Obstetrics, Gynecology & Women’s Health, University of Minnesota, Minneapolis, MN, USA; 5 Quantitative Biomedical Research Center, University of Texas Southwestern Medical Center, Dallas, TX, USA; 6 Department of Clinical Sciences, University of Texas Southwestern Medical Center, Dallas, TX, USA; 7 Simmons Cancer Center, University of Texas Southwestern Medical Center, Dallas, TX, USA

## Abstract

**Background:**

Many large-scale studies analyzed high-throughput genomic data to identify altered pathways essential to the development and progression of specific types of cancer. However, no previous study has been extended to provide a comprehensive analysis of pathways disrupted by copy number alterations across different human cancers. Towards this goal, we propose a network-based method to integrate copy number alteration data with human protein-protein interaction networks and pathway databases to identify pathways that are commonly disrupted in many different types of cancer.

**Results:**

We applied our approach to a data set of 2,172 cancer patients across 16 different types of cancers, and discovered a set of commonly disrupted pathways, which are likely essential for tumor formation in majority of the cancers. We also identified pathways that are only disrupted in specific cancer types, providing molecular markers for different human cancers. Analysis with independent microarray gene expression datasets confirms that the commonly disrupted pathways can be used to identify patient subgroups with significantly different survival outcomes. We also provide a network view of disrupted pathways to explain how copy number alterations affect pathways that regulate cell growth, cycle, and differentiation for tumorigenesis.

**Conclusions:**

In this work, we demonstrated that the network-based integrative analysis can help to identify pathways disrupted by copy number alterations across 16 types of human cancers, which are not readily identifiable by conventional overrepresentation-based and other pathway-based methods. All the results and source code are available at http://compbio.cs.umn.edu/NetPathID/.

## Background

Recent high-throughput technologies have enabled researchers to identify genomic alterations that could result in activation of oncogenes or inactivation of tumor suppressor genes, and thus disrupt pathways and biological processes known to contribute to tumor formation [1,2]. Many anticancer drugs have been developed to target proteins that act in these cancer-related pathways. Therefore, the precise identification and systemic characterization of altered activities in cancer-related pathways could accelerate the development of more effective targeted therapies, and aid in tailoring treatment to the genetic causes of an individual patient’s cancer [2].

Many large-scale genomic studies have been performed to define the cancer genome [3-11]. This effort is epitomized by The Cancer Genome Atlas [12-14] and its umbrella group, the International Cancer Genome Consortium [15]. Typically, in these studies, enrichment analysis was performed to identify statistically significant overlap between the list of altered genes and pathways or predefined gene sets [16-19]. For example, publications based on TCGA data have identified disrupted pathways in many cancer types, and these studies attempt to integrate sequence data, expression data, epigenetic data and copy-number data to get a wholistic view of the cancer genome [12-14].

In more advanced network analysis, altered genes (e.g. differentially expressed genes or mutated genes) are first projected onto an interaction network, and then clusters are found in this network. Ideker and colleagues pioneered this approach [16] and later extended the approach to identify network signatures (e.g. pathways, subnetworks, or functional modules) [20-27]. Similarly, pathway-based methods have been developed to incorporate interactions of member genes in known biological pathways to measure activities of pathways. These pathway-based methods were shown to be more accurate at identifying cancer-related pathways compared to overrepresentation-based enrichment analysis [22,28].

**Figure 1:**
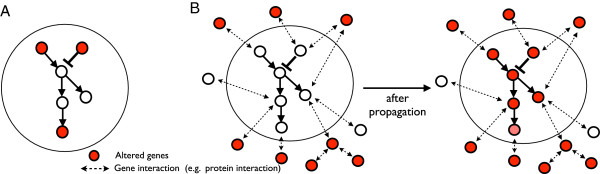
**Conceptual models for disrupted pathways.** This figure describes two conceptual models for inferring activity of disrupted pathways. **(A)** Three out of six member genes in the pathway are significantly altered by copy number changes. In this case, overrepresentation-based gene set enrichment analysis and pathway-based analysis could identify the pathway as an enriched pathway with altered genes, since many member genes in the pathway are altered in copy number changes. **(B)** No member gene in the pathway is altered by copy number changes, but member genes in the pathway are interacting with many other altered genes in the protein-protein interaction network. Existing gene set enrichment analysis and pathway-based analysis would fail to identify the pathway as a disrupted pathway, due to the lack of overlapping altered genes with member genes in the pathway. However, by applying a machine learning method, which propagates the activity score of genes to other genes by exploring cluster structures in the protein-protein interaction network, our approach could identify the pathway as a disrupted pathway, since many member genes in the pathway are interacting with other altered genes (i.e. significantly altered genes in copy number alterations could alter the activity (or function) of member genes through interactions).

A limitation is that these current methods are not designed to determine which pathways are disrupted in particular cancer types, and which are commonly disrupted across many types of human cancers. In this study, we describe an integrative network-based approach to identify pathways disrupted by copy number alterations in 2,172 cancer patients across 16 different types of cancers. Our approach is based on the assumption that copy number changes of a gene will affect the activity of the gene itself and the genes with which it interacts since amplification or deletion of genes could alter expression (or functions) of its neighbor genes in the networks [29]. We define a disrupted pathway as one whose members (genes) are directly altered, or they interact (based on the protein-protein interaction network) with many altered genes (Figure 1). Using an integrative analysis of copy number alterations and protein-protein interaction networks, our approach infers activity scores of all genes in the networks and makes use of inferred gene activity scores to identify pathways that are disrupted. Importantly, while overrepresentation-based enrichment analysis ignores altered genes not annotated in the specific pathway being analyzed, our method incorporates these genes using label propagation based on a protein-protein interaction database.

In the experiments, we first show the limitation of the enrichment analysis and current network-based analysis on DNA copy number, and then demonstrate that, although there are distinct patterns of copy number alteration in specific types of cancer, our method can identify common pathways disrupted in more than 10 different types of cancers. Our analysis of common and cancer-type specific disrupted pathways will lead to a better understanding of cancer network modules, and suggest potential therapeutic targets for cancer treatment. We also provide a network view of disrupted pathways to show how copy number alteration can disrupt core pathways that are essential for cancer development and progression.

## Results

### Limitation of enrichment analysis and current network-based analysis on DNA copy number alterations

Although many efforts have been made to build gene set databases (e.g. KEGG, Biocarta, Reactome, or the Gene Ontology database), and significant work has been done to expand the current knowledge of gene functions and roles in cellular systems, many human genes are not yet annotated in existing gene set databases. Most notably, we observed that more than 70% of the genes that are identified as significantly altered based on copy number alterations (see Methods) are not annotated in current pathway databases (Additional file 1: Figure S1). Due to this low coverage of gene annotation in significantly altered copy number regions, overrepresentation-based enrichment analyses, and standard pathway-based analyses are omitting some of the most significantly altered genes in their analyses. Hence, they provide a limited analysis of pathway activity that is based on the small non-representative fraction of altered genes that are currently annotated in pathway databases.

Previous network-based methods suffer less from incomplete gene annotations for inferring pathway activity; they also have difficulty in analyzing pathway activity across cancers. This is primarily due to the diverse copy number alteration patterns that exist in different human cancers. By performing copy number alteration analysis using Genomic Identification of Significant Targets in Cancer (GISTIC) [30] in 16 types of cancers, we have found a diverse spectra of copy number alteration patterns, and show that only a few significantly altered genomic regions are present across multiple cancer types (Additional file 1: Figure S2). This lack of coherence in copy number alterations across cancers could lead to the failure of some network-based methods to identify common biological pathways affected by copy number alterations. Our method introduced below overcomes this limitation by using a label propagation technique along with a protein-protein interaction network that includes these unannotated gene products.

### A network-based approach for discovering disrupted pathways based on copy number alterations across multiple cancer types

**Figure 2:**
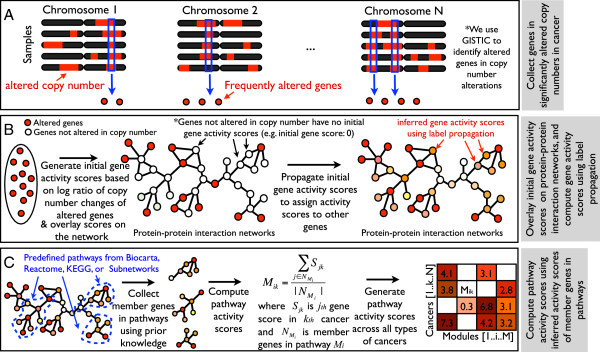
**Overview of NetPathID.** This figure describes steps to discover disrupted pathways across cancers. The aim of the approach is to integrate the copy number data with protein-protein interaction networks to quantify pathway activity for discovering disrupted pathways across cancers. **(A)** A list of significantly altered genes residing in copy number regions is generated using GISTIC. **(B)** We initialize activity scores of these genes using their average log2 ratios of amplification or deletion in copy number data, and overlay initial gene activity scores on the protein-protein interaction networks. To fully utilize network topological information, we apply a label propagation algorithm to assign gene activity scores to all the genes in the protein-protein interaction networks (see “Methods” section). **(C)** Finally, pathway activity scores are computed by average activity scores of member genes in each predefined pathway from prior knowledge (e.g. pathway database or conserved subnetworks in the protein-protein interaction networks cross species). We repeat step **(A)** and **(B)** to generate a matrix containing pathway activity scores from multiple cancer types.

We developed an algorithm called NetPathID (NETwork based method for PATHway IDentification) to discover pathways disrupted by copy number alterations across cancers (Figure 2). The aim of our approach is to integrate copy number changes of genes with the protein-protein interaction networks, and incorporate additional biological knowledge (e.g. pathway databases and conserved subnetworks across species) to discover disrupted pathways across human cancers. Our approach assumes that the activity of a pathway disrupted by copy number alterations can be quantified by the average of its member genes’ activity scores. Activity scores of genes are computed by a label propagation technique [31] that utilizes the global topological information in the protein-protein interaction network. This allows us to use genes/proteins of unknown function when initially assigning activity scores, and through label propagation, these scores will affect the activity scores of the annotated genes/proteins. The label propagation algorithm overlays the label information (i.e., activity score) on the vertices, and iteratively propagates scores among the neighboring vertices. The propagation process will finally converge toward the unique global optimum minimizing a quadratic criterion. Recently, label propagation and its variants have been successfully applied in many contexts including gene function prediction, disease gene prioritization, biomarker identification, and disease outcome prediction [31-36].

**Figure 3:**
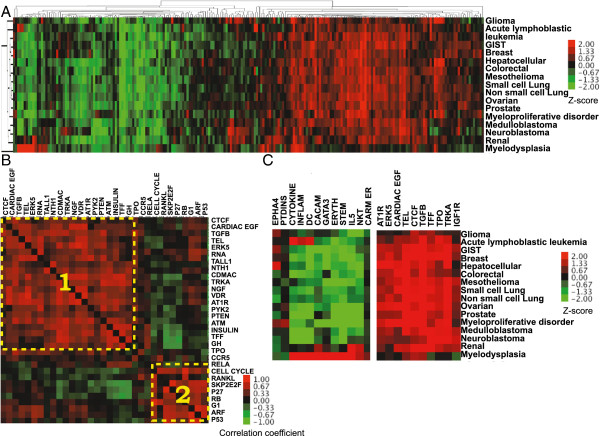
**Pathway activity view of cancers. (A)** Heat map describing the two-way hierarchical clustering of inferred activity of 217 Biocarta pathways across 16 types of cancers. Each row is a different type of cancer, and each column is a pathway. Color bar represents Z-score transformation of the activity score of the pathway. Red indicates significantly disrupted pathways, and green indicates pathways that are not disrupted by copy number alterations. **(B)** Heat map describing the correlation coefficient of pathway co-disruption (red: positive correlation, green negative correlation). The top 30 ranked disrupted pathways across cancers are included in the heat map. **(C)** Zoom-in plots including cancer-type specific and commonly disrupted pathways. For example, Cytokine, DC (“Dendritic cells in regulating TH1 and TH2 Development”), and INFLAM (“Cytokines and Inflammatory Response”) pathways are only disrupted in acute lymphoblastic leukemia and myelodysplasia. Cytokines and inflammatory response, as well as dendritic cells as modulators of immune responses in DC pathway are known for development of acute lymphoblastic leukemia and myelodysplasia. In contrast to cancer-type specific disrupted pathways, there is a set of commonly disrupted pathways across cancers. For example, TGFB (“TGF beta signaling”) pathway is one of commonly disrupted pathways across more than 10 types of cancers. Other commonly disrupted pathways include TEL (“Telomeres, Telomerase, Cellular Aging, and Immortality”), TRKA (or NTRK1) (“Trka Receptor Signaling Pathway”), CTCF (“First Multivalent Nuclear Factor”), and SPRY (“Sprouty regulation of tyrosine kinase signals”) pathways.

An illustration of NetPathID is provided in Figure 2. First, we collect a list of genes with significant copy number alterations in each type of cancer by using GISTIC. We use this list of altered genes to generate initial gene activity scores based on the log2 ratio values of copy number changes of altered genes (Step A in Figure 2). Second, we overlay initial gene activity scores on the protein-protein interaction networks, and apply label propagation to assign activity scores to all other genes in the protein-protein interaction networks. (Step B in Figure 2). Finally, we summarize computed activity scores of member genes in predefined pathways (or subnetworks) to identify altered activities of the pathways in each type of cancer (Step C in Figure 2). Larger activity scores indicate that the pathways are highly disrupted based on copy number alterations. We repeat these steps to identify altered activities of pathways for all 16 types of cancer. This allows us to generate a global map of pathway activity across cancers (Figure 3). We also provide a network view of disrupted pathways, which provides a wholistic impression of how copy number changes influence core pathways essential for the development and progress of cancers.

We performed extensive evaluation of NetPathID by comparing it with overrepresentation-based gene set enrichment analysis using hypergeometric testing, the method of Lee *et al.*[27,28] (Additional file 2). We found that NetPathID can accurately identify cancer-related pathways from negative controls (i.e., randomly generated decoy pathways). We also confirmed that commonly disrupted pathways identified by NetPathID are related to cancer biology (see Additional file 2). Finally, we found that NetPathID is robust with regards to the bias in the protein-protein interaction networks (see Additional file 2).

### Patterns of disrupted pathways based on copy number alterations across cancers

We applied our approach to copy number data from 16 types of human cancers, using information from protein-protein interaction network and predefined annotated pathway databases (see Methods). Statistical significance of the pathway score was assessed through permutation (Additional file 1: Figure S7). Pathways with significant Benjamini- Hochberg adjusted p-values were selected by using a false discovery rate cutoff of 0.005 for Biocarta, and 0.10 for both the KEGG and Reactome databases. In addition, to determine if a pathway was significantly disrupted in the given cancer type, we further filtered the selected pathways that were ranked in the top 20% based on pathway activity scores in each cancer type. Finally, 488, 456, and 855 (14%, 15%, and 12% of the total) pathways from Biocarta, KEGG, and Reactome pathway databases, respectively, were found to be significant across 16 types of cancers, and used for further analysis.

### Co-disruption of pathways by copy number alterations in human cancers

Biological pathways often function cooperatively to contribute to phenotypes such as cancer. Thus, advances in understanding these pathways and their interconnectivity will accelerate the development of molecular targeted therapies that promise to change the practice of oncology [37]. We first explored patterns of disrupted pathways by using two-way hierarchical clustering to identify clusters of pathways that are statistically co-disrupted (Figure 3A). Our analyses identified clear relationships among disrupted pathways such as telomerase (TEL), TGF-beta, RB, and P53 pathways. For example, the telomerase pathway is co-disrupted with TGF-beta, ATM signaling, and CTCF: First Multivalent Nuclear Factor (CTCF) pathways (Figure 3B (1)). A recent study experimentally validated that the TGF-beta signaling pathway negatively regulates the telomerase pathway, and other studies also reported that MDM2, which is a gene in the ATM pathway, and CTCF both inhibit the expression of telomerase [38-40]. Another interesting example of co-disrupted pathways includes ARF, p53, and RB pathways (Figure 3B (2)). Disruption of the RB pathways could activate the ARF pathway, and the activation of ARF could trigger the p53 pathway, which induces growth arrest and/or apoptosis [41]. These consistent observations with previous studies demonstrate that NetPathID is capable of identifying valid patterns of co-disrupted pathways. Thus, further analysis of co-disrupted pathways may help to provide novel insights into the nature of pathway associations.

### Common and cancer-type specific disrupted pathways based on copy number changes across cancers

We further attempted to identify common pathways disrupted by copy number alterations across cancers. We used the following strict criteria to define commonly disrupted pathways: 1) Pathways must have significant BH-adjusted p-values; 2) Pathways must be ranked within the top 10% compared to other pathways in each cancer study based on their activity scores, and; 3) Top ranked pathways must be present in at least 10 different types of cancer. Based on these criteria we found an average of 14 commonly disrupted pathways from KEGG, Biocarta, and Reactome (Figure 3, and Additional file 3: Tables S1 and S2). Examples of commonly disrupted pathways from the Biocarta pathway database include telomerase, transforming growth factor beta (TGF-Beta) signaling pathway, NTRK1 (TrkA) signaling and Cell Cycle pathways. Some of these pathways were already known to be altered broadly across many cancer types, such as TGF-Beta [42], Cell Cycle [43] and telomerase [44]. Other pathways, though, have only been implicated in a few cancers. For example, TrkA signaling is known to play a role in neuronal cancers as well as a few non-neuronal cancers such as medullary thyroid carcinoma, lung, pancreatic, ovarian and breast cancers [45]. However, TrkA signaling has not been functionally associated with cancers such as ALL, GIST, mesothelioma and renal cancer, which were identified in our analysis. This could have important implications because therapeutics have been developed that target TrkA signaling [46].

As we expected, NetPathID identified cancer related pathways such as the KEGG annotated Pancreatic, Colorectal, Glioma, Lung, Prostate and Bladder cancer pathways. Likewise, using the Reactome annotated pathways our method identified well known cancer-related pathways that regulate cell growth, death and proliferation including the EGFR signaling pathway, NF-kb activation, and Ras signaling pathway as commonly disrupted across many types of cancers (Additional file 3: Table S1). Importantly, our method also identified pathways that were not previously considered to be universally disrupted in cancers, such as the Adherens Junction and PECAM1 pathways [47]. Our analysis found these pathways to be significant in all 16 cancer types in the KEGG annotated pathways and Reactome annotated pathways (Additional file 3: Table S1). These results support the idea of using novel targeted therapies, such as the monoclonal antibody targeting PECAM1 [48], in a wider array of cancer types.

In addition to pathways disrupted in the majority of cancers, we also found that there are sets of pathways that are only disrupted in specific types of human cancers (Figure 3C). Some examples include Cytokine Network (cytokines), Cytokines and Inflammatory Response (INFLAM), Dendritic cells in regulating TH1 and TH2 Development (DC) pathways, which are all only disrupted in acute lymphoblastic leukemia and myelodysplasia. These disrupted pathways are widely involved in T cell and B cell activities that are associated with immune responses, and activation and proliferation of specific differentiated immune cells. Other cancer-type specific disrupted pathways include the Sonic Hedgehog/Patched1 (SHH) Receptor Ptc1 Regulates cell cycle (PTC) pathway disrupted in renal and glioma cancers, and Role of Ran in mitotic spindle regulation (RANMS) pathway disrupted in colorectal cancer (Additional file 3: Table S13). These findings are supported by functional studies which have demonstrated that inhibition of the Sonic Hedgehog signaling pathway is known to induce renal cancer, and enhances the efficacy of targeted therapy in glioma [49,50]. Ran, which controls the cell cycle through the regulation of mitotic spindle organization, was shown to be highly expressed in many cancer types including gastric and colorectal, and is known for its involvement in malignant transformation and/or the enhanced proliferation of cancer cells [51].

These observations suggest that commonly disrupted pathways across many types of cancers could play a major role in the development of cancers, while the set of disrupted pathways that are specific to certain types of cancers could help to characterize these types of cancers and provide options for different targeted therapies.

Current pathway databases cover only a small fraction of human genes. Therefore, although the use of these pathway databases as prior knowledge helps to define and identify disrupted pathways, it is possible that there are many more gene modules as yet undescribed that contribute to cancer. To tackle this challenge, we obtained 4,620 protein-protein interaction subnetwork modules that are conserved across different species, and use them as additional pathway data [52]. The conserved subnetworks were identified by PathBLAST [53] among two (or more) species, and cover more than 8,558 proteins (genes). We used NetPathID with these 4,620 subnetwork modules and identified 41 commonly disrupted subnetworks that are present in at least 10 types of cancer and are ranked within top 5% in each cancer study (see Additional file 3: Table S1). This nicely illustrates how NetPathID is not simply biased to genes in existing databases, and is able to highlight modules of uncharacterized genes that are worthy of further study.

### Commonly disrupted pathways across cancers correlate with clinical outcomes

We investigated whether we could use disrupted pathways discovered by NetPathID to identify subgroups of patients that correlate with different clinical outcomes, such as survival. Specifically, we hypothesized that commonly disrupted pathways reflect molecular mechanisms contributing to the biological/clinical behavior of cancers. Thus, we could use member genes in disrupted pathways as gene signatures to identify patient subgroups having different clinical outcomes.

**Figure 4:**
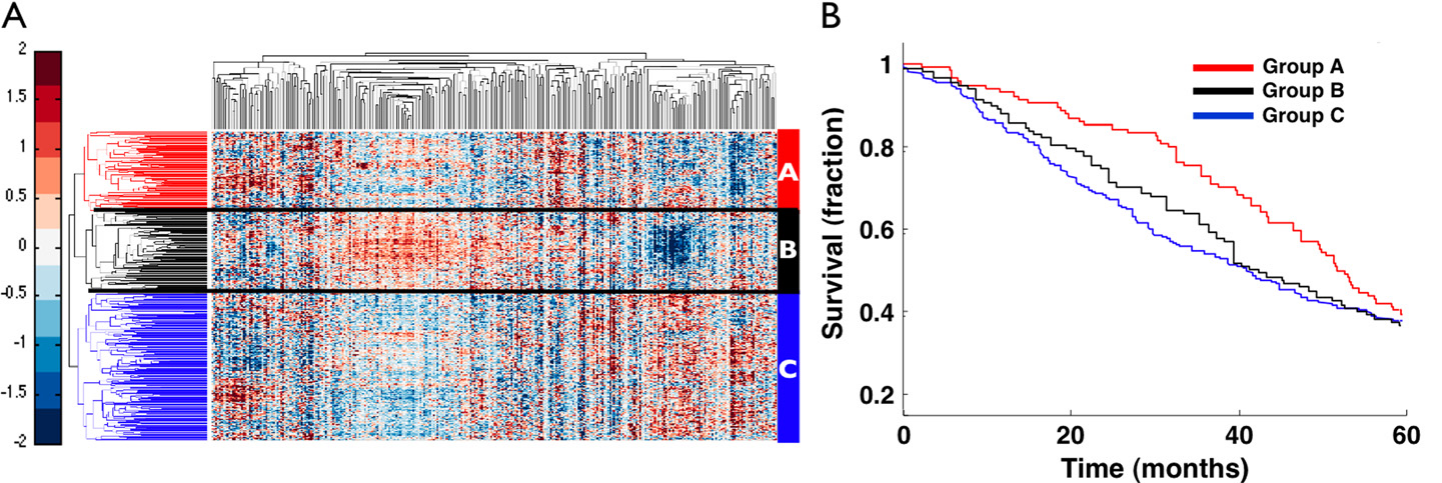
**Commonly disrupted pathways across cancers correlate with clinical outcomes. (A)** Two-way hierarchical clustering of lung cancer patients using member genes in commonly disrupted pathways. **(B)** Kaplan-Meier survival plots for the clusters of patient subgroups from lung cancer microarray gene expression dataset. Colors (Red, Black and Blue) indicate patient subgroups used for Kaplan-Meier analysis.

To test our hypothesis we collected gene expression data with clinical information from four independent microarray gene expression datasets [54-57]. The genes used for clustering were the set of 331 genes in the 42 commonly disrupted subnetworks identified by NetPathID. We identified patient subgroups by visual examination of the clustering results, and generated Kaplan-Meier curves for the subgroups (Figure 4). In the lung cancer data set [56], we found three patient groups, with group C patients having significantly worse survival outcomes than group A (logrank test p-value < 0.0000198, Hazard ratio = 1.4910), with a median survival time of 40 months for group A and 23 months for group C. Similarly, in a breast cancer data set and two ovarian cancer data sets, we were able to partition the patients into different groups using the same gene set, and noted that these patient groups have significantly different survival profiles (Additional file 1: Figure S9). These results suggest that the analysis of top ranked disrupted pathways may allow stratification of cancers at the pathway level, which could have prognostic value and possibly aid in diagnosis and treatment decisions.

### Cancer-related genes are enriched in commonly disrupted pathways

We also investigated the commonly disrupted pathways in terms of their enrichment for known cancer-related genes. We hypothesized that commonly disrupted pathways across many types of cancers have many cancer “driver” genes as key components. More specifically, the presence of known cancer-causing genes with well-defined biological properties in a functional module can be used to make reasonable guesses about other candidate “driver” genes in the same functional module. To validate our hypothesis, we defined a functional module as the union of our commonly disrupted pathways (i.e., ranked within top *k*% in each cancer study, and present in more than *10* cancer types), and computed the fraction of known cancer-causing genes based on the Cancer Gene Census database from the Sanger Institute in the functional module. A higher cancer gene fraction for the functional module indicates more enrichment for cancer-causing genes. For comparison, we use the Lee *et al.* method [22,28] that overlays gene scores obtained from aggregated and pooled analyses to pathways, and then ranked them to identify the top-ranked disrupted pathways.

**Figure 5:**
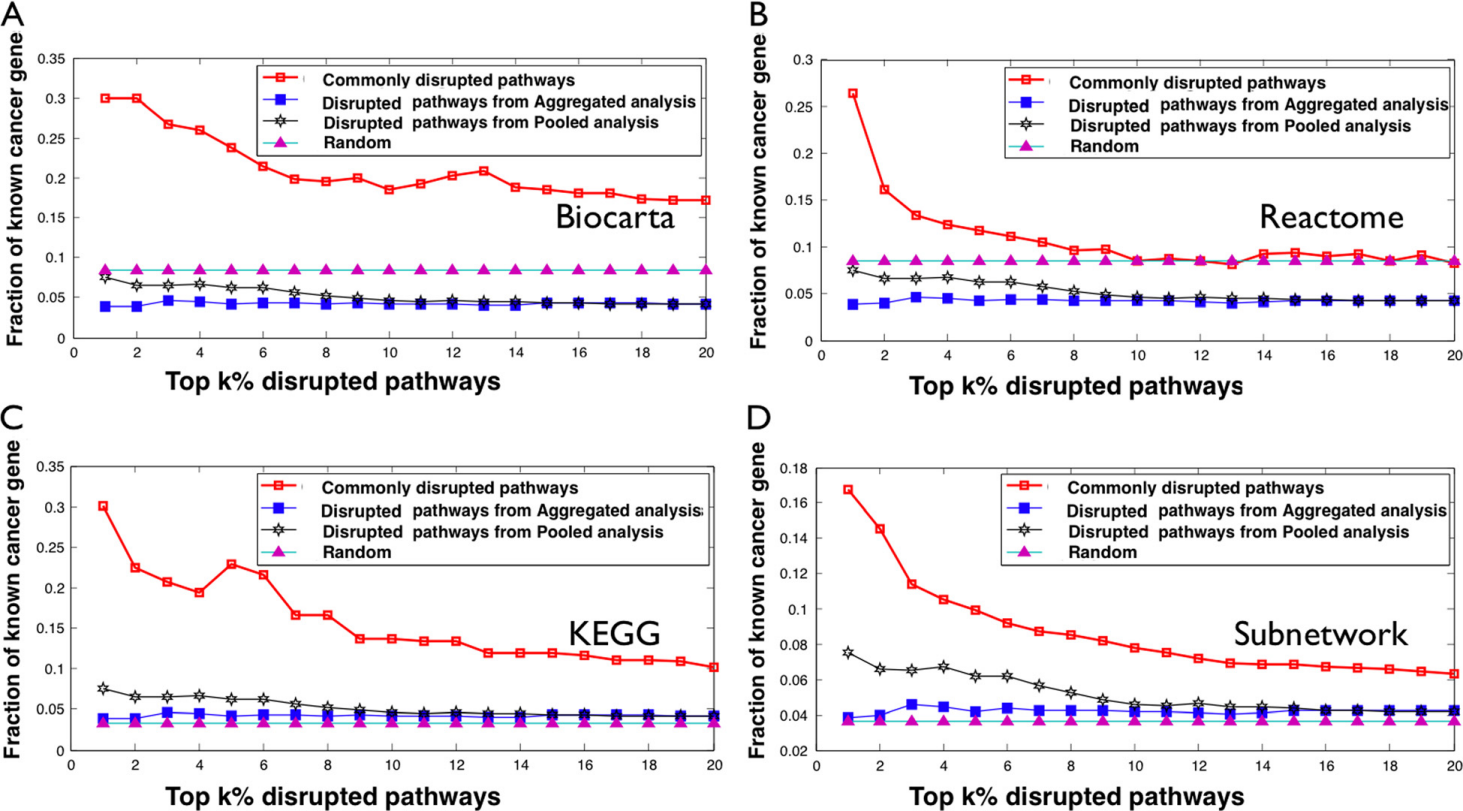
**Cancer-related genes are enriched in commonly disrupted pathways.** Fraction of known cancer genes in top k% ranked disrupted pathways based on pathway activity score using pathway information from **(A)** Biocarta, **(B)** Reactome, **(C)** KEGG, and **(D)** conserved protein-protein interaction subnetworks.

We observed a significantly higher fraction of known cancer-causing genes in functional modules from our commonly disrupted pathways and subnetworks than random, aggregated or pooled analyses. On average, the fraction of known cancer genes was 2-fold higher than the control groups. For example, Figure 5(A) shows the fraction of known cancer causing genes in the functional modules from the Biocarta pathway database. Reassuringly, the fraction of known cancer genes in our functional modules were consistently higher than the fraction in the two baseline datasets when analyzing the top 1 to 5% of the disrupted pathways (see Additional file 2). In three of four of our functional modules, the fraction remained higher even when including up to 20% of the top disrupted pathways.

The higher fraction of known cancer-causing genes in functional modules from our commonly disrupted pathways is consistent with our hypothesis that commonly disrupted pathways across many types of cancers would have many cancer “driver” genes. Thus, we predict that the other genes in these functional modules could also be cancer “drivers” and warrant further study.

### An example of a disrupted signaling pathway: TGF-beta signaling pathway

**Figure 6:**
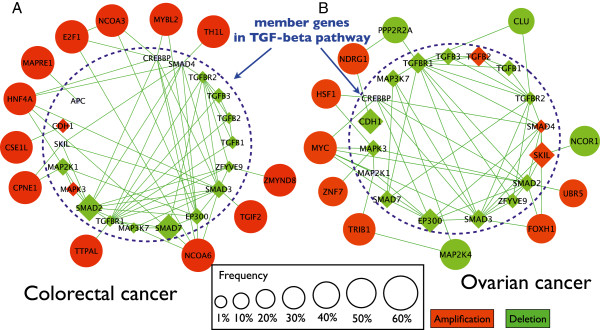
**Network view of TGF-beta signaling pathway alterations in colorectal and ovarian cancer. (A)** Network view of genes altered by copy number changes in colorectal cancer in the TGF-beta signaling pathway (diamond nodes) or genes directly interacting with TGF-beta signaling genes (circular nodes) based on the protein-protein interaction database. **(B)** The same network view for ovarian cancer. Size of node represents frequency of amplification or deletion in patient population. Color of node indicates whether gene is amplified (red), deleted (green), or unchanged (gray). Lines indicate interactions. Blue dotted line separates genes within the pathway from genes that interact with the pathway based on the protein-protein interaction database.

Using NetPathID, we identified the TGF-beta signaling pathway as one of the commonly disrupted pathways from the Biocarta, KEGG, and Reactome pathway databases. To visualize our findings, we generated a network view of the TGF-beta signaling pathway from Biocarta, and displayed the pathway in colorectal cancer and ovarian cancer in Figure 6. We also present a network view of the TGF-beta signaling pathway across all cancers (Additional file 1: Figure S3).

It is evident from the network visualization that many of the annotated genes in TGF-beta signaling pathway have a relatively low recurrent frequency, which would imply that the pathway is only disrupted in a small cohort of cancer patients. However, by including interacting genes it becomes apparent that many genes that directly interact with members of the TGF-beta signaling pathway are altered in a significant percentage of cancer types. We also noted that the interacting partners are different for different cancer types. For example, in colorectal cancer, MAPRE1 binds to the tumor suppressor protein APC which is often mutated in familial and sporadic forms of colorectal cancer. MAPRE1 is also involved in processes including cell migration and adhesion, transcriptional activation, and apoptosis. In our data, the copy number of APC is not significantly altered by copy number, but our network view indicates that amplification of MAPRE1 could affect pathways and processes involved in APC regulation. In a similar manner, other highly amplified genes that are not annotated with the TGF-beta signaling pathway are interacting with genes in the TGF-beta signaling pathway, and thus, those highly amplified genes could affect activity of the TGF-beta signaling pathway. Another example is the relationship between HNF4a and colorectal cancer (Additional file 1: Figure S13a). HNF4A, a transcription factor regulated by TGF-beta signaling [58], is associated with diabetes and HCC, but has only recently been linked to intestinal tract pathology including ulcerative colitis [59] and Crohn’s disease [60]. Our results indicate it may also be playing a role in colorectal cancer,

In ovarian cancer, the annotated genes in the TGF-beta pathway also have a relatively low recurrent frequency, while genes that directly interact with these annotated genes are altered in a high percentage of cancers. Interestingly, the interacting genes are not the same interacting genes found in the colorectal cancer network. In ovarian cancer, a few of the interesting interacting genes include MYC, a well-known oncogene and TRIB1, a novel regulator of the MAP kinase pathway recently linked to leukemogenesis [61].

These network views provide biologically meaningful insights into how copy number alterations in different genes, among different types of cancers could affect common pathways. The illustrations also demonstrate the usefulness of an integrative analysis to discover disrupted pathways, which contain many member genes each having low significance with respect to copy number changes.

## Discussion

Despite the success of our approach, there are limitations to the method. First, we use both amplified and deleted genes without distinguishing the two types. Thus, it would not be straightforward to interpret the effect on the activity of the pathways across cancers, because the disruption may be caused by either amplified or deleted genes. It would be possible to extend the method by separating amplification and deletion, but this could limit the ability to identify pathways with both amplified and deleted genes. In fact, we found many pathways disrupted by both amplified and deleted genes. Thus, one promising direction for further improvement of the method would be to incorporate other complementary genomic datasets to determine the role of disrupted pathways. For example, we could include datasets measuring expression of genes downstream of the pathways to determine the effect of copy number alterations on the pathways.

Another limitation in our analysis is the relatively sparse coverage of current protein-protein interaction databases. Instead of using the protein-protein interaction databases we could, instead, use functional linkage networks, which have more comprehensive coverage of a broad variety of gene relationships, and could allow for more sensitive discovery of network signatures under various conditions of interest [62]. While existing network-based methods cannot handle the high density of these large-scale functional networks, the label propagation-based methods were successfully applied to functional linkage networks in a recent study [62]. Thus, it would be interesting to use functional linkage networks to discover robust and reliable disrupted pathways across cancers.

As with all studies that use annotated pathways, there is the problem of overlap between pathways and the decision to include/exclude proteins when the pathway is annotated. For example, ERK1/2 and AKT are members of many of the annotated pathways such as TGF-beta, TRKA, and Telomerase that were identified in our study, yet the pathways specifically named “MAPK” or “AKT” by the annotating organization did not register as significantly altered. Our findings suggest that these pathways as a whole are not commonly dysregulated, but only specific aspects of these pathways are co-opted by cancer.

In this work, we have presented analysis using input from GISTIC with default settings. To test the robustness of NetPathID we tested different cutoff levels using GISTIC to see if the list of disrupted pathways would change. We found that NetPathID was remarkably robust even when the cutoff values were raised from the default setting of 0.1 to 0.3 or 0.5. Most of the rankings of commonly disrupted pathways and the rankings of pathways that were specific to one type of cancer remained constant (see Additional file 2 and Additional file 3: Tables S15–S21).

Another possible bias could arise from the inclusion of whole chromosome arm deletions or amplifications (e.g., 11q is clearly lost for neuroblastoma in Additional file 1: Figure S3), because it is likely that the majority of the genes on the chromosome arm are not driving tumor growth. We adapted NetPathID so that we could run the analysis with or without including whole chromosome arm gains and losses. Again, surprisingly, most of the rankings did not change significantly (see Additional file 2 and Additional file 3: Tables S15–S21).

One possible explanation for the robustness of NetPathID is that NetPathID is not limited to the set of genes in the genomic regions detected by GISTIC. The gene set is expanded using our label propagation method which results in pathway activity scores based on a larger gene set. In addition, NetPathID is initiated using the average log2 ratio of the amplified or deleted gene detected by GISTIC. Therefore, genes with low log2 ratios will have less of an effect than genes with high log2 ratios.

Finally, although our study focused on the discovery of disrupted pathways from datasets of copy number alterations, the algorithm is general and can be readily applied to other types of genomic data, including, gene expression, mutation, and methylation.

## Conclusions

We have described a network-based integrative method for discovering disrupted pathways based on copy number alterations in human cancers. NetPathID integrates copy number data and the protein-protein interaction networks to quantify activity scores of pathways. Specifically, NetPathID effectively utilizes information in the protein-protein interaction network and copy number changes with label propagation to quantify altered activities of pathways. This approach has the potential to uncover disrupted pathways that cannot be discovered by using overrepresentation and pathways-based methods, which rely on a limited number of annotated genes. Thus, NetPathID is uniquely suitable for providing a global analysis of disrupted pathways across cancers.

We applied our approach to copy number data from 16 types of cancers, and discovered commonly disrupted pathways and pathways that are only disrupted in specific types of cancer. Functional enrichment analysis of commonly disrupted pathways demonstrated that many cancer types share common biological processes that define the malignant state, including self-sufficiency in growth signaling, insensitivity to antigrowth signals, inactivation of apoptosis, and genomic instability. Of particular significance, we identified a patient subpopulation with poor survival using member genes in disrupted pathways, implying the potential of these disrupted pathways to serve as a guide to therapy in a subgroup of patients.

## Methods

### Copy number data preparation

The copy number data from 16 human cancer types were collected from a recent study after removing cell lines and datasets with fewer than 15 patients samples (Sept. 2010) [63] (http://www.broadinstitute.org/tumorscape/pages/portalHome.jsf). Copy-number measurements were obtained using the Affymetrix 250 K SNP arrays. For the details of preprocessing and segmentation of copy number dataset, please refer to [63]. To detect significantly altered copy number regions, we use GISTIC with default settings, with exceptions indicated in the text [30].

### Human protein-protein interaction and pathway data

We obtained the protein-protein interaction network from the Human Protein Reference Database (May 2010) [64]. This network contained 9,667 proteins and 76,132 binary edges. We obtained KEGG, Biocarta, and Reactome gene sets from MsigDB (Sept. 2010) [18] and 4,620 conserved subnetworks in the human protein-protein interaction network from [52]. To reduce bias to disease proteins in the protein-protein interaction network, we use the extended protein-protein interaction network suggested by [65]. The extended protein-protein interaction network is generated by combining the HPRD, OPHID, BIND, and MINT database, and has a similar degree of interactions for both disease and non-disease proteins. For details of network preparation and statistics, please refer to [65].

### NetPathID algorithm

The algorithm identifies pathways disrupted by genes with copy number alterations. Disrupted pathways are found based on high pathway activity scores across cancers. There are three main steps in NetPathID:

1. Collecting altered genes based on copy number alterations

Our approach requires lists of genes with copy number alterations, and corresponding gene scores representing the log2 ratio of copy number changes. We use GISTIC as a filter to identify recurrently altered regions in each type of cancer. The genes within these regions define a seed gene set of frequently amplified and deleted genes, and *G*_*amp*_ where *g*_*i*_ is a frequently amplified or deleted gene if *g*_*i*_ ∈*G*_*amp*_ or *G*_*del*_, respectively.

2. Computing gene score

After collecting a seed gene set based on GISTIC, the next step is to compute gene activity scores. We use a label propagation algorithm to compute gene scores, and this label propagation takes two inputs: 1) an adjacency matrix describing the gene network, and 2) an initial gene activity score vector. We define the adjacency matrix of the gene network to be *G*_(*n*×*n*)_ where *n* is the number of genes in the protein-protein interaction networks. We generate an initial gene score vector *g*  =  [*g*_1_,  *g*_2_, …,  *g*_*n*_]^*T*^ denoting the average log ratio of a frequently amplified or deleted gene in each dataset, where $g_{i}\;=\;\frac{\sum_{j=1}^{m}S_{i,j_{\mathrm{amp}}}}{m}$ if *g*_*i*_  ∈ *G*_*amp*_, or $g_{i}\;=\;\frac{\sum_{j=1}^{m}\left|S_{i,j_{\mathrm{del}}}\right|}{m}$ if *g*_*i*_  ∈ *G*_*del*_, otherwise *g*_*i*_ = 0, and *n* and *m* represent the number of genes and patients in dataset, and $S_{i,j_{\mathrm{amp}}}$, and $S_{i,j_{\mathrm{del}}}$ represent log ratio of an amplified and deleted gene *g*_*i*_ in *j* th patient in dataset. Specifically, we compute the average log2 ratio of each amplified or deleted gene across patient samples to use it as an initial gene activity score to compute its final gene activity score. To fully utilize the network topological information to compute the final gene activity score, we generate $\bar{G}$, the graph Laplacian of the gene network *G*_(*n*×*n*)_, to use to propagate initial gene activity scores to genes in the network. Here $\bar{G}\;=\;D_{G}^{-\frac{1}{2}}GD_{G}^{-\frac{1}{2}}$, and *D*_*G*_ is a diagonal matrix with diagonal elements *D*_*G*_  =   ∑ _*j*_*G*_*i*,*j*_. A vector $\tilde{g}$ for final gene activity scores is derived from the following optimization problem [35].

(1) $${\text{min}}_{\tilde{g}}\sum_{i,j}{\bar{G}}_{i,j}{\left({\tilde{g}}_{i}-{\tilde{g}}_{j}\right)}^{2}\;+\;\frac{1-\alpha }{\alpha }\sum_{i}{\left({\tilde{g}}_{i}-g_{i}\right)}^{2}$$

In equation (1), the first term is a smoothness penalty, which forces connected genes to receive similar activity scores, and the second term ensures the consistency with the initial gene scores. Label propagation combines the neighboring information in the network with the consistency with the initial gene activity scores to provide global activity scores to genes in the network. Parameter *α* ∈(0,1) balances contributions from two penalties. Note that we use 0.5 for our parameter in this work (see Additional file 1: Figure S11 for the effects of different parameter choices of the alpha). The closed form solution of equation (1) is $\tilde{g}\;=\;\left(1-\alpha \right){\left(I-\alpha \bar{G}\right)}^{-1}g$. Empirically, to avoid computing the inverse of $\left(I-\alpha \bar{G}\right)$, an iterative algorithm can efficiently compute the closed-form solution with the following update rule at each time step *t*,

$${\tilde{g}}^{t}\;=\;\left(1-\alpha \right)g\;+\;\alpha \bar{G}{\tilde{g}}^{t-1}.$$

3. Computing the pathway activity score

After computing activity scores of genes in each type of cancer, we summarize activity scores of the member genes in the pathways to compute activity scores of pathways as

$$\mathrm{Pathway}\;\mathrm{score}\;=\;M_{\mathrm{jk}}\;=\;\frac{\sum_{i\in N_{\mathrm{Mj}}}{\tilde{g}}_{\mathrm{ik}}}{\left|N_{\mathrm{Mj}}\right|},$$

where *M*_*jk*_ is activity score of the *j*th pathway in *k*th type of cancers, and *N*_*Mj*_ is the member genes in pathway *M*_*j*_[52].

### Pooled and aggregated analysis

To perform the pooled analysis, we first incorporate all of the copy number data from 2,172 patients into one dataset. Then, we run GISTIC to identify the set of genes with significant copy number alterations. To discover disrupted pathways enriched with this set of genes, we rank genes based on –log10(qval) from GISTIC, and then select the top k% genes to perform overrepresentation-based analysis using hypergeometric testing. To perform the aggregated analysis, we run GISTIC to calculate the significance of the altered genes in each cancer type. After running GISTIC on all datasets, we summarize –log10(qval) for each gene across all datasets. Then we rank genes based on aggregated –log10(qval), and select the top k% genes to perform overrepresentation-based analysis using hypergeometric testing. Note that if one gene has a qval from GISTIC for both amplification and deletion, we select the more significant qval for the gene.

### Significance of pathway scores

To assess significance of the pathway score, we performed the analysis on random datasets. To construct these control datasets we randomly shuffled initial gene activity scores and pathway member assignments 10,000 times to generate a background distribution of pathway scores. From this control dataset we were able to derive the empirical p-value of the actual scores.

### Analysis of patterns of disrupted pathways

In each cancer type, pathway activity scores were Z-score transformed. Then, we perform two-way hierarchical clustering using Cluster 3 with complete linkage to analyze patterns of pathway co-disruption based on the inferred pathway activity for each cancer type in the dataset. To validate the correlation between clustered pathways, we use the Matlab corrcoeff function. Then, two-way hierarchical clustering was performed to plot the heat map describing the correlation coefficient of pathway co-disruption.

### Network view of disrupted pathways

To generate the network view of the pathway, we collected annotated genes in the pathway, and genes that directly interact with one of the annotated genes based on the protein-protein interaction database. Interacting genes were only included in the network if their GISTIC-q-value was among the top 500 values out of 18,932 ranked genes.

### Microarray gene expression data preparation, and clustering

Four microarray gene expression datasets were used for the identification of patient subgroups. The lung cancer dataset was downloaded from [56]. We downloaded two ovarian cancer datasets (GSE9891, GSE3149) and one breast cancer dataset (GSE2034) from Gene Expression Omnibus (GEO). All datasets were RMA normalized, log transformed, and expression values were median centered. To perform unsupervised hierarchical clustering, we use Matlab clustergram function with average linkage.

## Competing interests

THH, GA, RK, KATS, VK, and TS have no competing interests. PMH, ZZ, and JL are employees and stockholder of Genentech Inc./F. Hoffmann-La Roche Ltd.

## Authors’ contributions

THH designed the study, performed the statistical analysis, and drafted the manuscript. GA performed the statistical analysis. RK conceived of the study, and helped to draft the manuscript. VK conceived of the study, and helped to draft the manuscript. TS performed the analysis and significant manuscript editing and revisions. KATS performed significant manuscript editing and revisions. PMH conceived of the study, and participated in the design of the study. ZZ conceived of the study, and participated in the design of the study. JL conceived of the study, designed the study, supervised the study, performed the analysis, and drafted the manuscript. All authors read and approved the final manuscript.

## Supplementary Material

Additional file 1: Figure S1 The fraction of annotated genes in copy number alterations. **Figure S2.** The significance of copy number alterations across cancers. **Figure S3.** TGF--‒beta signaling pathway is commonly disrupted by genes in copy number alterations across multiple types of cancers. **Figure S4.** Telomerase pathway is commonly disrupted by genes in copy number alterations across multiple types of cancers. **Figure S5**. NTRK1 (TrkA) signaling pathway is commonly disrupted by genes in copy number alterations across multiple types of cancers. **Figure S6.** Distribution of pathway activity scores. **Figure S7.** Heat map describing the correlation coefficient of pathway co--‒disruption. **Figure S8.** False discovery rate using decoy pathways. **Figure S9.** Clustering and Kaplan--‒Meier analysis. **Figure S10.** A functional map of commonly disrupted pathways across cancers. **Figure S11.** Effects on different parameter choices of alpha. Click here for file

Additional file 2 **Comparisons with Lee *et al.* method and overrepresentation-based enrichment methods, and HotNet.** Click here for file

Additional file 3: Table S1 Commonly disrupted pathways using NetPathID. **Table S2.** Disrupted pathways using aggregated and pooled analysis. **Table S3.** Baseline comparision. **Table S4.** Commonly disrupted pathways using extended PPI network. **Table S5.** GO biological process enriched with genes in commonly disrupted pathways from Biocarta pathway database. **Table S6.** GO molecular function enriched with genes in commonly disrupted pathways from Biocarta pathway database. **Table S7.** GO biological process enriched with genes in commonly disrupted pathways from Reatome pathway database. **Table S8.** GO molecular function enriched with genes in commonly disrupted pathways from Reactome pathway database. **Table S9.** GO biological process enriched with genes in commonly disrupted pathways from KEGG pathway database. **Table S10.** GO molecular function enriched with genes in commonly disrupted pathways from KEGG pathway database. **Table S11.** GO biological process enriched with genes in commonly disrupted pathways from conserved subnetwork modules. **Table S12.** GO molecular function enriched with genes in commonly disrupted pathways from conserved subnetwork modules. **Table S13.** Top ranked disrupted pathways by all the methods from Biocarta pathway database. **Table S14.** Data statistics for each cancer type. **Table S15.** Commonly disrupted pathways using GISTIC with different cutoffs. **Table S16.** Top ranked disrupted pathways by NetPathID with different GISTIC cutoffs from Biocarta pathway database. **Table S17.** Data statistics for top ranked disrupted pathways from GISTIC with different cutoffs. **Table S18.** Data statistics for # genes detected by GISTIC with different cutoffs. **Table S19.** Commonly disrupted pathways before vs after arm-level copy number alterations. **Table S20.** Top ranked disrupted pathways by NetPathID with after removing arm-level copy number alterations. **Table S21.** Data statistics for top ranked disrupted pathways from before and after removing arm-level copy number alterations. Click here for file
