# Supplementary material for: Large-scale integrative network-based analysis identifies common pathways disrupted by copy number alterations across cancers
Source: BMC Genomics. 2013 Jul 3;14:440. doi: 10.1186/1471-2164-14-440 (PMC3703268; doi:10.1186/1471-2164-14-440)
Supplement: Additional file 1: Figure S1 — The fraction of annotated genes in copy number alterations. Figure S2. The significance of copy number alterations across cancers. Figure S3. TGF--‒beta signaling pathway is commonly disrupted by genes in copy number alterations across multiple types of cancers. Figure S4. Telomerase pathway is commonly disrupted by genes in copy number alterations across multiple types of cancers. Figure S5. NTRK1 (TrkA) signaling pathway is commonly disrupted by genes in copy number alterations across multiple types of cancers. Figure S6. Distribution of pathway activity scores. Figure S7. Heat map describing the correlation coefficient of pathway co--‒disruption. Figure S8. False discovery rate using decoy pathways. Figure S9. Clustering and Kaplan--‒Meier analysis. Figure S10. A functional map of commonly disrupted pathways across cancers. Figure S11. Effects on different parameter choices of alpha. [file 1471-2164-14-440-S1.pdf]

[Supplementary Figure] Large-scale integrative network-based analysis identifies common pathways disrupted by copy number alterations across cancers

TaeHyun Hwang†[1], Gowtham Atluri[2], Rui Kuang[2], Vipin Kumar[2], Timothy Starr[1,4], Kevin Silverstein[1], Peter M Haverty[3], Zemin Zhang[3], Jinfeng Liu†[3]

[1]Masonic Cancer Center, [2]Department of Computer Science and Engineering, University of Minnesota - Twin Cities, Minneapolis, Minnesota, United States of America; [3]Department of Bioinformatics and Computational Biology, Genentech Inc., South San Francisco, California, United States of America; [4]Department of Obstetrics, Gynecology & Women's Health, University of Minnesota, Minneapolis, Minnesota, United States of America

† To whom correspondence should be addressed. E-mail: (hwang071@umn.edu, liu.jinfeng@gene.com)

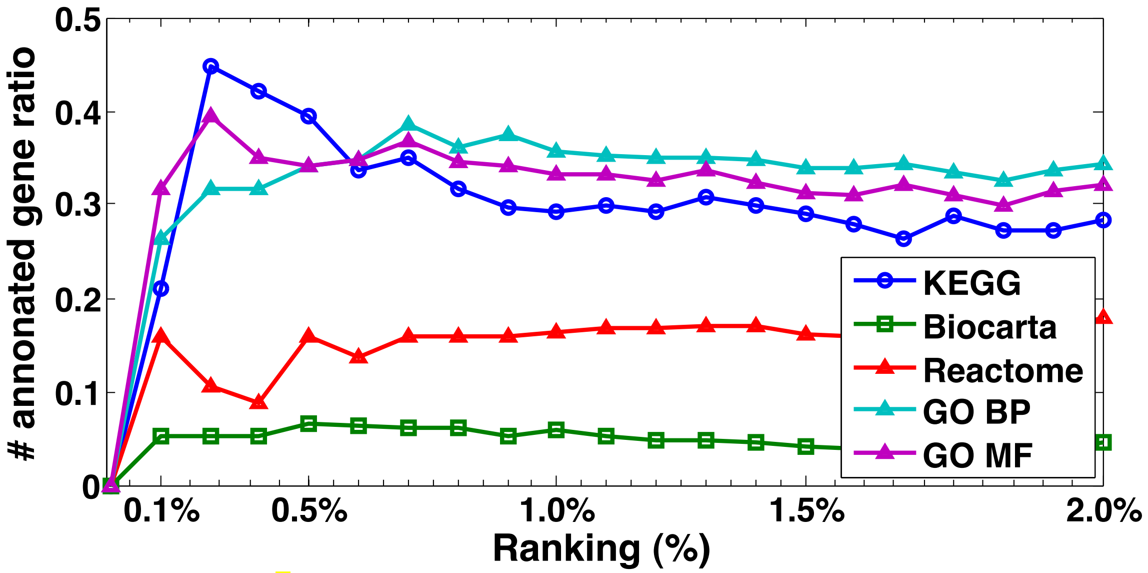

Supplementary Figure 1. The fraction of annotated genes in copy number alterations This figure describes the fraction of significantly altered genes in copy number that are annotated with existing pathway and process database. Each gene in copy number alteration is ranked by its corresponding qval from GISTIC using pooled analysis. For example, more than 230 out of 380 (top 2%) ranked genes (18,932 genes in hg18) in significantly altered copy number regions are not annotated with KEGG, Biocarta, Reactome, GO Biological Process (BP), and Molecular function (MF).

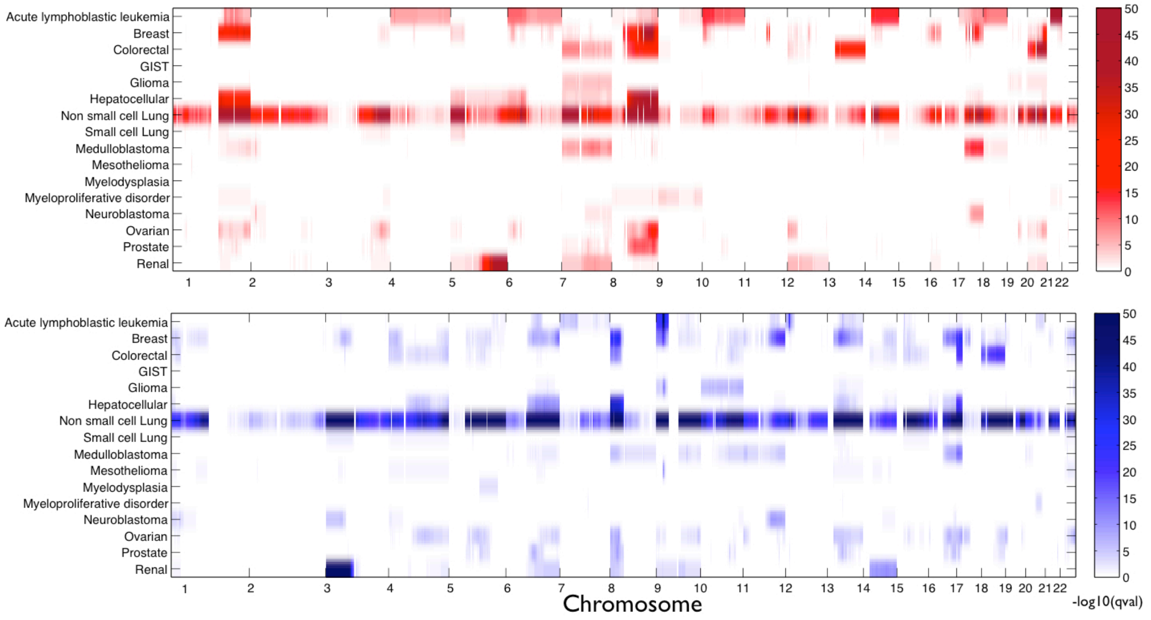

Supplementary Figure 2. The significance of copy number alterations across cancers This figure describes the significance of amplifications (top, red), and deletions (bottom, blue) on genomes (x-axis) across different types of cancers (y-axis) using GISTIC-qval. Qval from GISTIC is -log10 transformed.

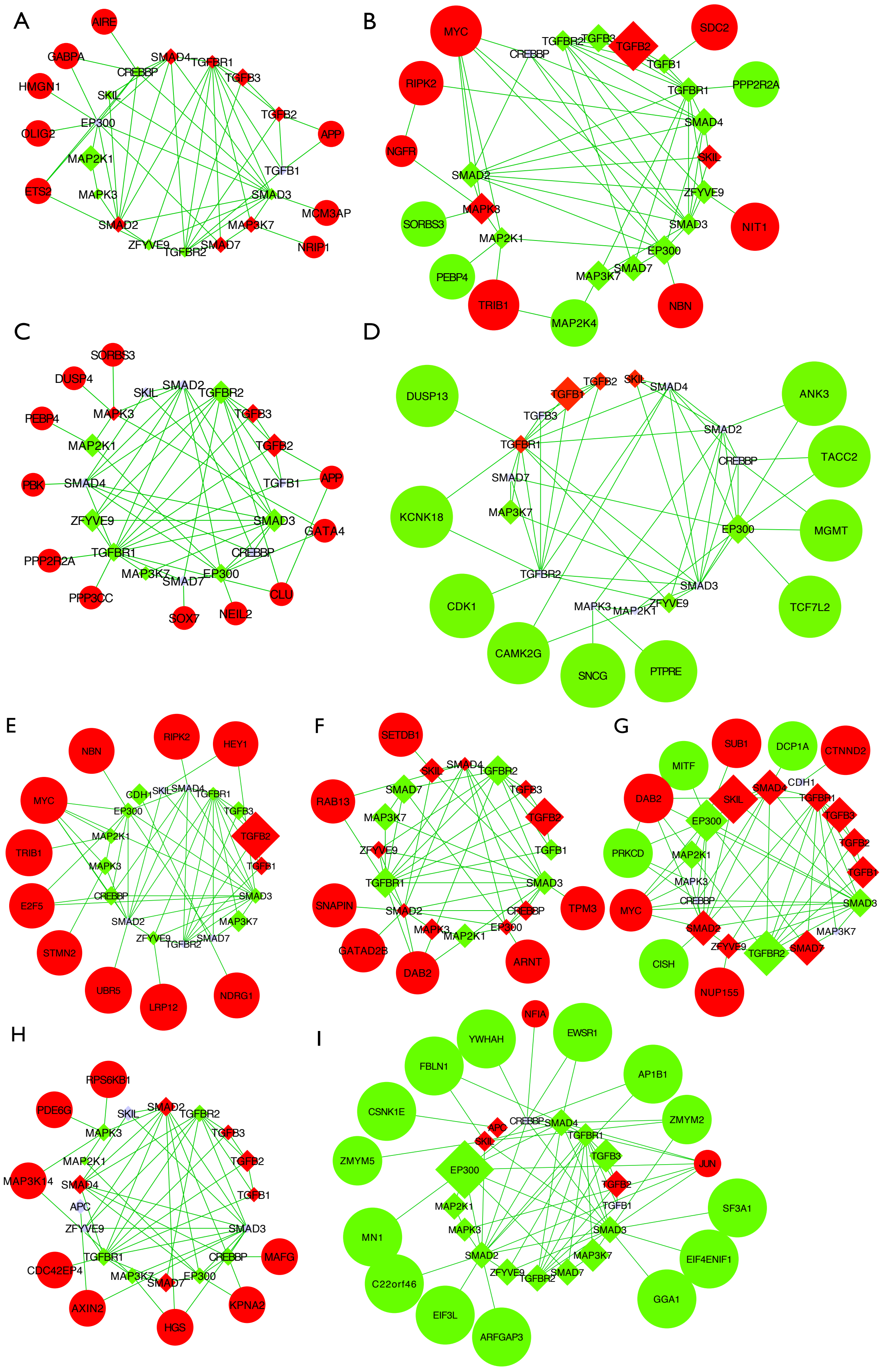

A
AIRE
GABPA
SMAD4
TGFBR1
CREBBP
TGFB3
HMGN1
SKIL
EP300
TGFB2
OLIG2
APP
MAP2K1
TGFB1
ETS2
MAPK3
SMAD3
MCM3AP
SMAD2
MAP3K7
ZFYVE9
SMAD7
TGFBR2
NRIP1
B
MYC
TGFBR2
TGFB3
TGFB2
SDC2
CREBBP
TGFB1
RIPK2
TGFBR1
PPP2R2A
SMAD4
NGFR
SKIL
SMAD2
ZFYVE9
MAPK8
SORBS3
SMAD3
NIT1
MAP2K1
EP300
SMAD7
PEBP4
MAP3K7
TRIB1
NBN
MAP2K4
C
SORBS3
DUSP4
SMAD2
SKIL
TGFBR2
MAPK3
TGFB3
PEBP4
MAP2K1
TGFB2
APP
TGFB1
PBK
SMAD4
ZFYVE9
SMAD3
GATA4
TGFBR1
CREBBP
PPP2R2A
MAP3K7
EP300
SMAD7
CLU
PPP3CC
SOX7
NEIL2
D
DUSP13
TGFB1
TGFB2
SKIL
SMAD4
ANK3
TGFB3
SMAD2
TGFBR1
CREBBP
TACC2
SMAD7
KCNK18
MAP3K7
EP300
MGMT
TGFBR2
SMAD3
CDK1
MAPK3
MAP2K1
ZFYVE9
TCF7L2
CAMK2G
SNCG
PTPRE
E
RIPK2
HEY1
NBN
CDH1
SKIL
SMAD4
TGFBR1
MYC
EP300
TGFB3
TGFB2
MAP2K1
TRIB1
MAPK3
TGFB1
CREBBP
SMAD3
E2F5
SMAD2
MAP3K7
ZFYVE9
SMAD7
TGFBR2
STMN2
UBR5
LRP12
NDRG1
F
SETDB1
SKIL
SMAD4
TGFBR2
SMAD7
TGFB3
RAB13
MAP3K7
TGFB2
ZFYVE9
TGFB1
TGFBR1
SMAD3
SNAPIN
SMAD2
CREBBP
TPM3
MAPK3
MAP2K1
EP300
GATAD2B
DAB2
ARNT
G
SUB1
DCP1A
CTNND2
MITF
DAB2
SKIL
SMAD4
CDH1
TGFBR1
TGFB3
EP300
PRKCD
MAP2K1
TGFB2
MAPK3
TGFB1
CREBBP
MYC
SMAD3
SMAD2
MAP3K7
ZFYVE9
TGFBR2
SMAD7
CISH
NUP155
H
RPS6KB1
PDE6G
SKIL
SMAD2
TGFBR2
MAPK3
TGFB3
MAP2K1
TGFB2
MAP3K14
SMAD4
TGFB1
APC
ZFYVE9
SMAD3
TGFBR1
CREBBP
MAFG
CDC42EP4
MAP3K7
SMAD7
EP300
AXIN2
KPNA2
HGS
I
NFIA
YWHAH
EWSR1
FBLN1
AP1B1
CSNK1E
APC
CREBBP
SMAD4
ZMYM2
SKIL
TGFBR1
ZMYM5
TGFB3
JUN
EP300
TGFB2
TGFB1
SF3A1
MAP2K1
MAPK3
SMAD3
MN1
MAP3K7
SMAD2
EIF4ENIF1
ZFYVE9
TGFBR2
SMAD7
C22orf46
GGA1
EIF3L
ARFGAP3

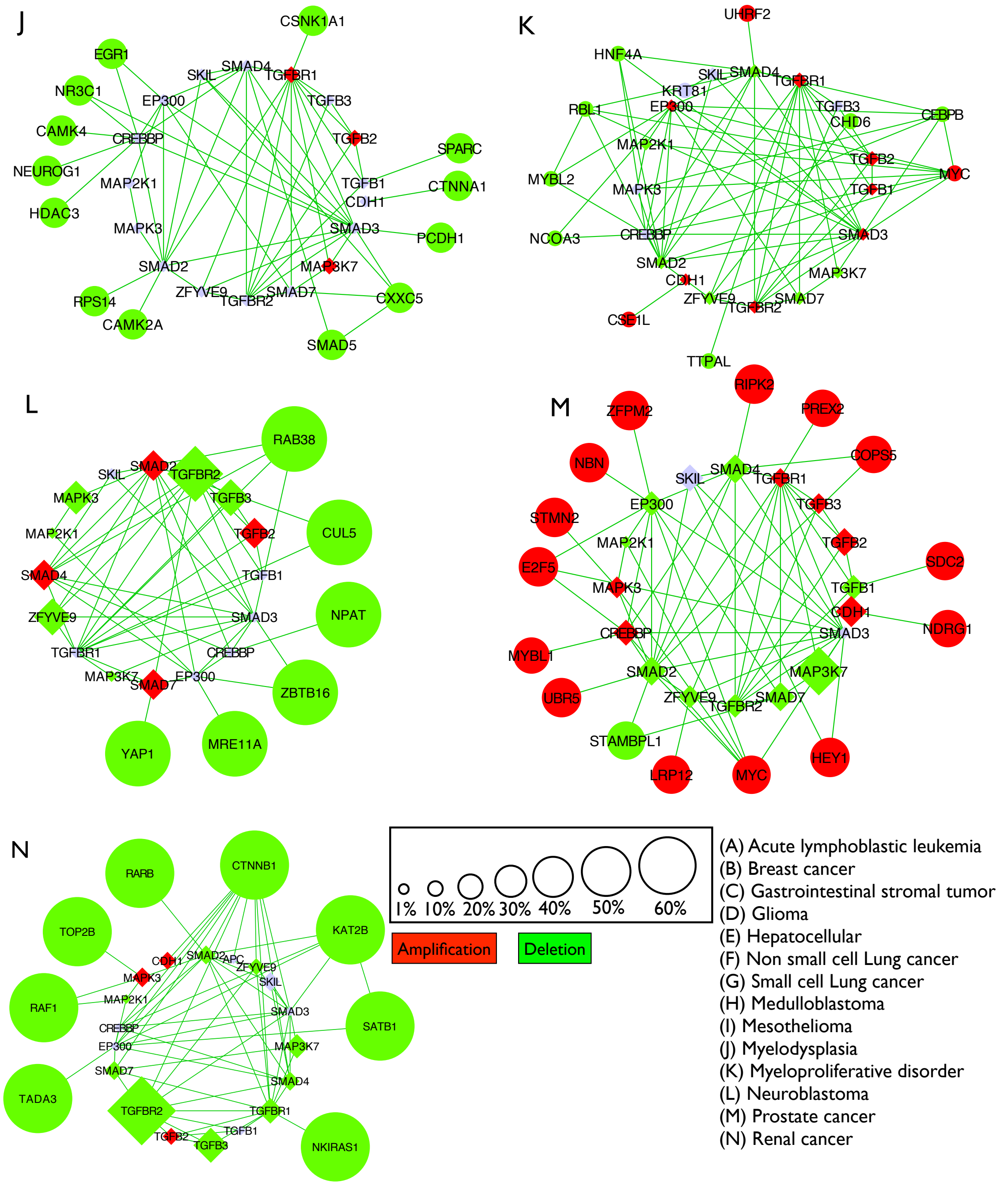

Supplementary Figure 3. TGF-beta signaling pathway is commonly disrupted by genes in copy number alterations across multiple types of cancers. This figure describes commonly disrupted TGF-beta signaling pathway across many cancer types. Red, and green colors indicate that genes are amplified, or deleted in copy number alterations, respectively. Gray color indicates that the gene is not altered by copy numbers. Diamond shape node represents that member genes in the pathway, and circular shape node represents genes that are interacting with member genes in the pathway in protein-protein interaction network. Size of nodes represents recurrent frequency of genes in copy number data (i.e. larger size of gene node indicates the gene is

frequently altered in patient samples). One interesting observation is that copy number changes of genes in the TGF-beta signaling pathway are different across many types of cancers, but they are consistently interacting with frequently altered genes in the protein-protein interaction network.

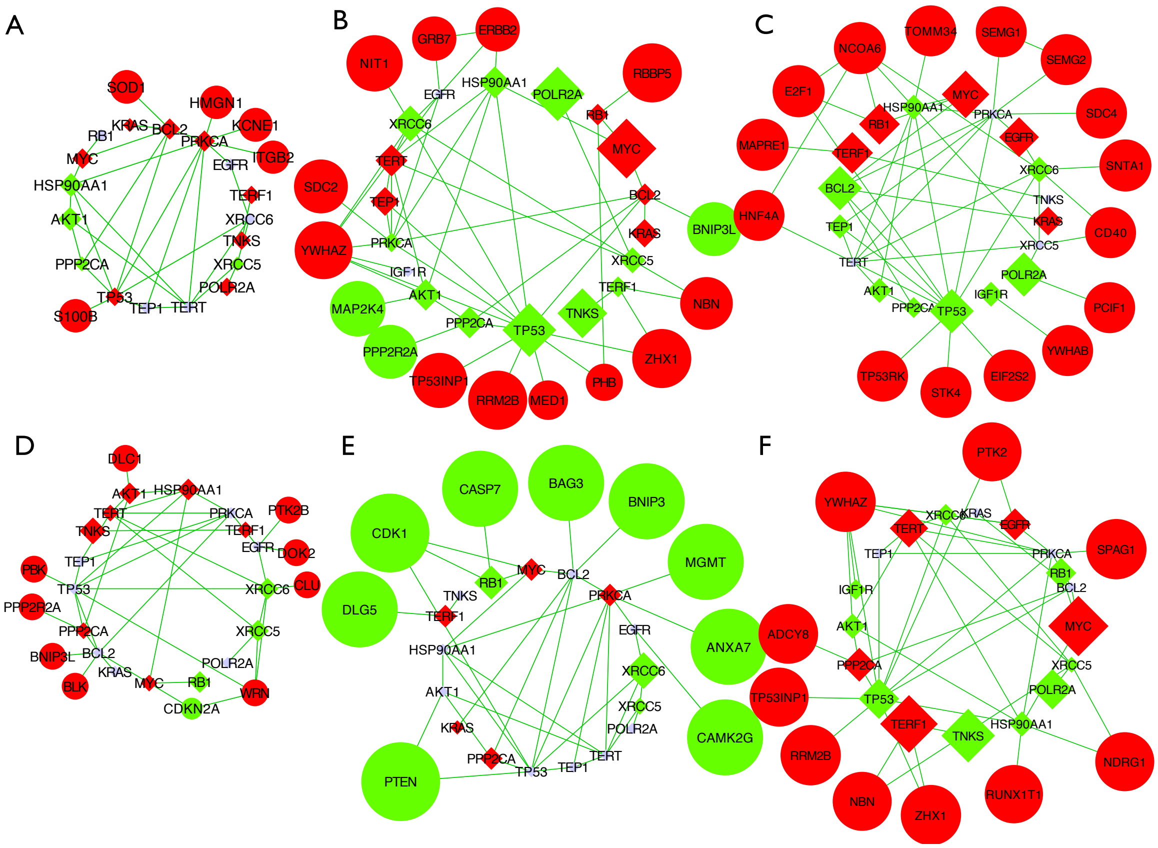

A
SOD1
HMGN1
KCNE1
ITGB2
RB1
KRAS
BCL2
PRKCA
MYC
EGFR
HSP90AA1
TERF1
XRCC6
AKT1
TNKS
XRCC5
PPP2CA
POLR2A
TP53
S100B
TEP1
TERT
B
GRB7
ERBB2
NIT1
HSP90AA1
EGFR
POLR2A
RBBP5
XRCC6
RB1
MYC
TERT
SDC2
TEP1
BCL2
PRKCA
KRAS
YWHAZ
IGF1R
XRCC5
BNIP3L
TERF1
AKT1
MAP2K4
NBN
PPP2CA
TNKS
TP53
PPP2R2A
ZHX1
TP53INP1
RRM2B
MED1
PHB
C
TOMM34
SEMG1
NCOA6
SEMG2
E2F1
HSP90AA1
MYC
RB1
PRKCA
SDC4
MAPRE1
EGFR
TERF1
XRCC6
SNTA1
BCL2
TNKS
HNF4A
KRAS
TEP1
CD40
XRCC5
TERT
POLR2A
AKT1
IGF1R
PPP2CA
TP53
PCIF1
YWHAB
TP53RK
STK4
EIF2S2
D
DLC1
AKT1
HSP90AA1
TERT
PRKCA
PTK2B
TNKS
TERF1
EGFR
DOK2
TEP1
PBK
CLU
TP53
XRCC6
PPP2R2A
PPP2CA
XRCC5
BCL2
BNIP3L
POLR2A
KRAS
MYC
RB1
WRN
BLK
CDKN2A
E
CASP7
BAG3
BNIP3
CDK1
MGMT
MYC
RB1
BCL2
TNKS
PRKCA
DLG5
TERF1
EGFR
ANXA7
HSP90AA1
XRCC6
AKT1
XRCC5
KRAS
POLR2A
CAMK2G
TERT
PPP2CA
TEP1
TP53
PTEN
F
PTK2
YWHAZ
KRAS
TERT
EGFR
SPAG1
TEP1
PRKCA
RB1
IGF1R
BCL2
MYC
AKT1
ADCY8
XRCC5
PPP2CA
POLR2A
TP53INP1
TP53
TERF1
TNKS
HSP90AA1
RRM2B
NDRG1
RUNX1T1
NBN
ZHX1

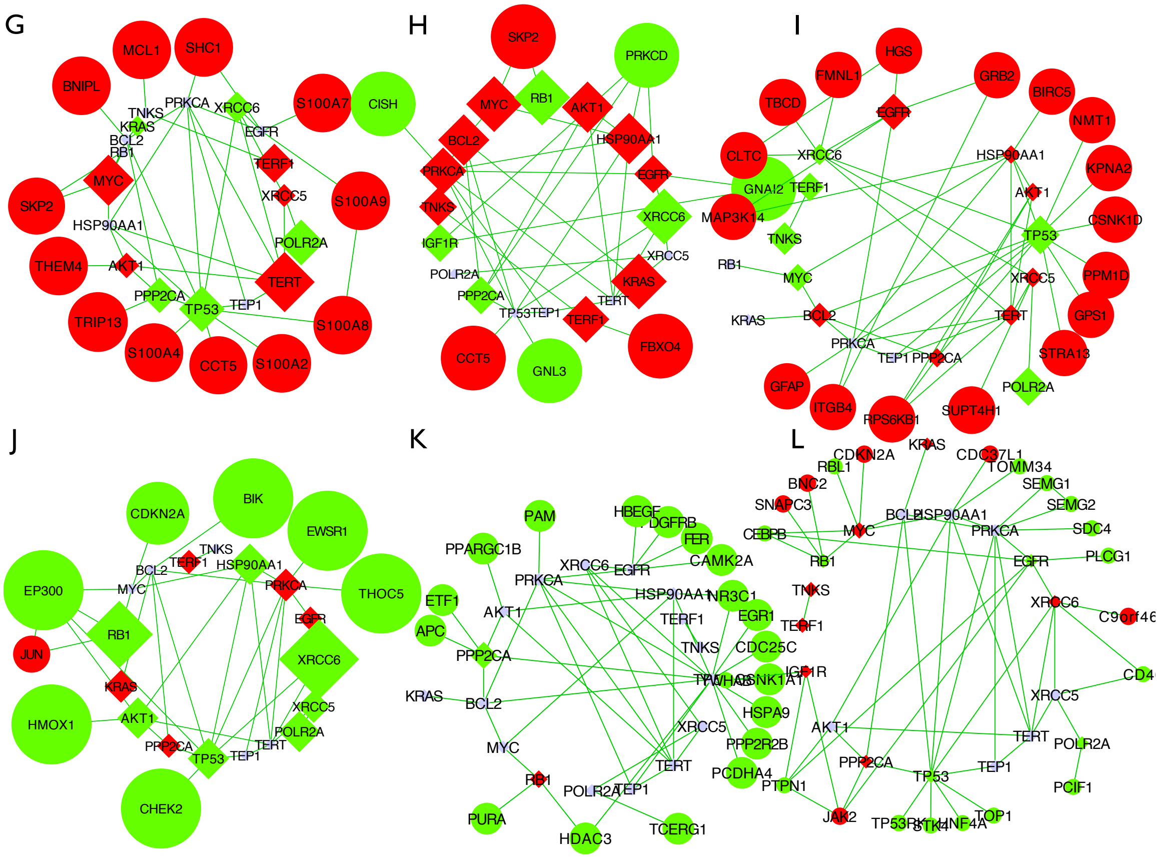

G
MCL1
SHC1
BNIPL
TNKS
PRKCA
XRCC6
S100A7
CISH
KRAS
BCL2
EGFR
RB1
TERF1
MYC
SKP2
XRCC5
S100A9
HSP90AA1
POLR2A
THEM4
AKT1
TERT
PPP2CA
TRIP13
TP53
TEP1
S100A8
S100A4
CCT5
S100A2
H
SKP2
PRKCD
MYC
RB1
AKT1
BCL2
HSP90AA1
PRKCA
EGFR
TNKS
XRCC6
IGF1R
XRCC5
POLR2A
KRAS
PPP2CA
TERT
TP53TEP1
TERF1
CCT5
GNL3
FBXO4
I
HGS
FMNL1
GRB2
TBCD
BIRC5
EGFR
NMT1
CLTC
XRCC6
HSP90AA1
KPNA2
GNAI2
TERF1
AKT1
MAP3K14
CSNK1D
TNKS
TP53
RB1
MYC
XRCC5
PPM1D
KRAS
BCL2
TERT
GPS1
PRKCA
TEP1
PPP2CA
STRA13
GFAP
POLR2A
ITGB4
RPS6KB1
SUPT4H1
J
BIK
CDKN2A
EWSR1
TNKS
TERF1
HSP90AA1
BCL2
EP300
MYC
PRKCA
THOC5
EGFR
RB1
JUN
XRCC6
KRAS
XRCC5
HMOX1
AKT1
POLR2A
TERT
PPP2CA
TP53
TEP1
CHEK2
K
PAM
HBEGF
PPARGC1B
FER
CAMK2A
XRCC6
EGFR
PRKCA
HSP90AA1
NR3C1
ETF1
AKT1
EGR1
TERF1
APC
TNKS
CDC25C
PPP2CA
KRAS
BCL2
HSPA9
XRCC5
MYC
PPP2R2B
TERT
PCDHA4
RB1
POLR2A
TEP1
PURA
HDAC3
TCERG1
L
KRAS
CDKN2A
RBL1
TOMM34
BNC2
SEMG1
SNAPC3
SEMG2
HSP90AA1
CEBPB
MYC
PRKCA
SDC4
RB1
EGFR
PLCG1
TNKS
XRCC6
TERF1
IGF1R
CD4
XRCC5
AKT1
TERT
POLR2A
PPP2CA
TP53
TEP1
PTPN1
PCIF1
JAK2
TOP1

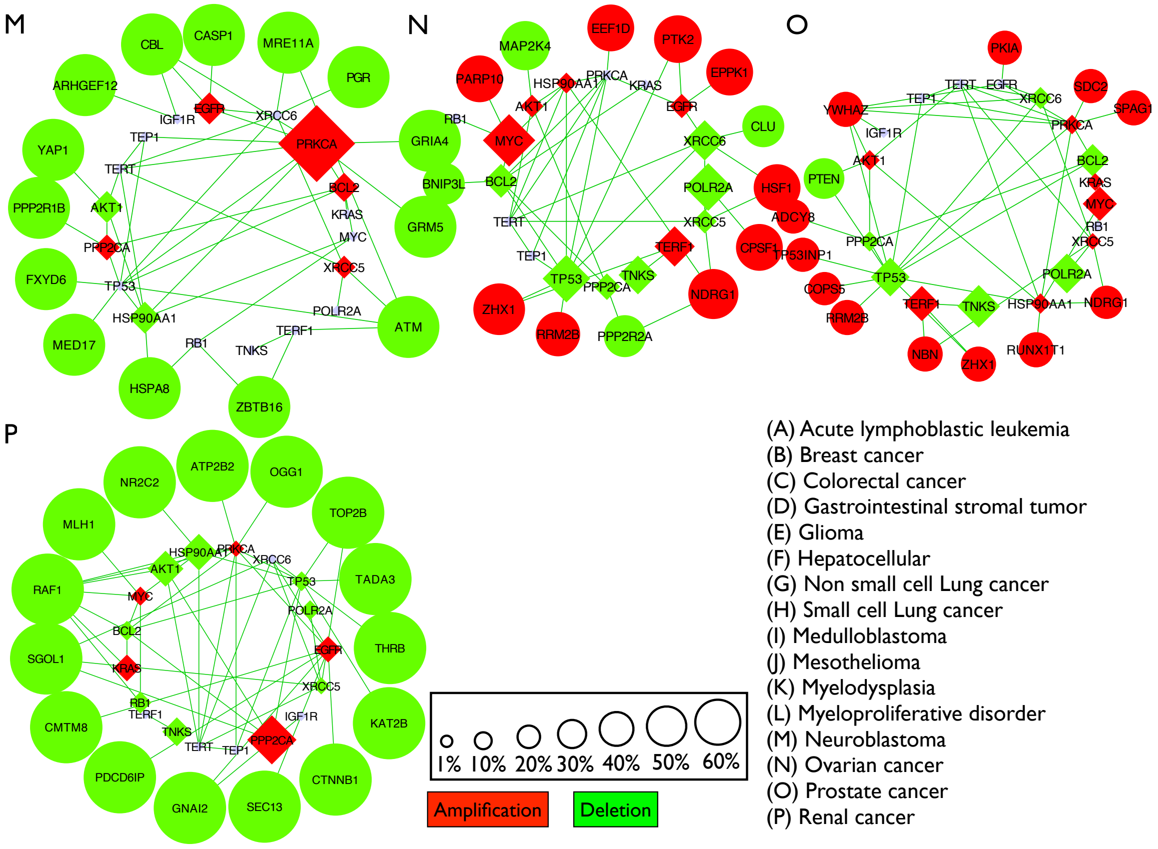

Supplementary Figure 4. Telomerase pathway is commonly disrupted by genes in copy number alterations across multiple types of cancers. This figure describes commonly disrupted Telomerase pathway across many cancer types. Red, and green colors indicate that genes are amplified, or deleted in copy number alterations, respectively. Gray color indicates that the gene is not altered by copy numbers. Diamond shape node represents that member genes in the pathway, and circular shape node represents genes that are interacting with member genes in the pathway in protein-protein interaction network. Size of nodes represents recurrent frequency of genes in copy number data (i.e. larger size of gene node indicates the gene is frequently altered in patient samples).

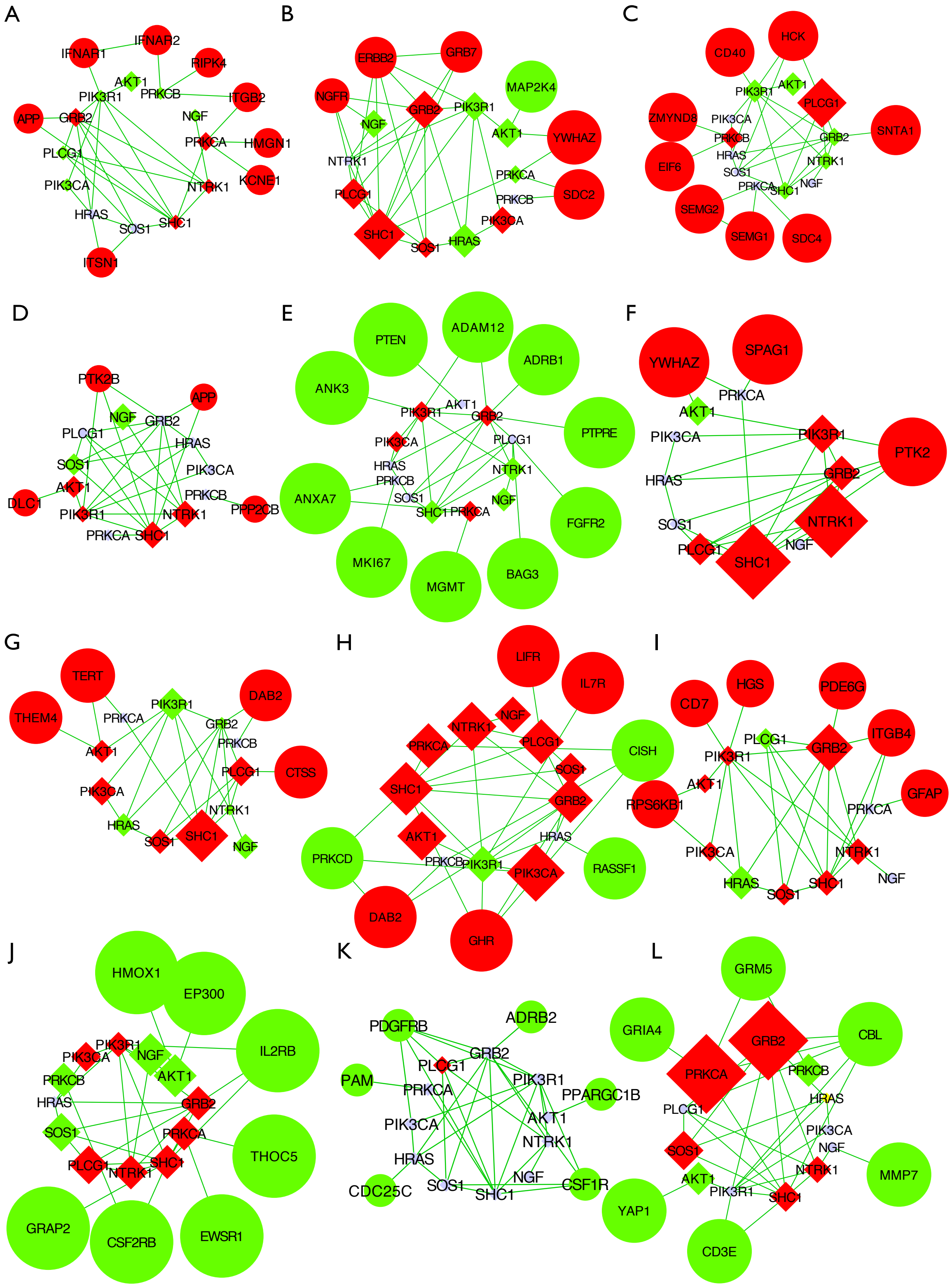
A
IFNAR1
IFNAR2
RIPK4
AKT1
PIK3R1
PRKCB
ITGB2
APP
GRB2
NGF
PRKCA
HMGN1
PLCG1
PIK3CA
NTRK1
KCNE1
HRAS
SOS1
SHC1
ITSN1
B
ERBB2
GRB7
MAP2K4
NGFR
GRB2
PIK3R1
NGF
AKT1
YWHAZ
NTRK1
PRKCA
PLCG1
PRKCB
SDC2
PIK3CA
SHC1
SOS1
HRAS
C
HCK
CD40
AKT1
PIK3R1
PLCG1
ZMYND8
PIK3CA
SNTA1
PRKCB
GRB2
HRAS
EIF6
SOS1
NTRK1
PRKCA
SHC1
NGF
SEMG2
SEMG1
SDC4
D
PTK2B
APP
NGF
GRB2
PLCG1
HRAS
SOS1
PIK3CA
AKT1
PRKCB
DLC1
PIK3R1
NTRK1
PPP2CB
PRKCA
SHC1
E
ADAM12
PTEN
ADRB1
ANK3
AKT1
PIK3R1
GRB2
PTPRE
PIK3CA
PLCG1
HRAS
NTRK1
PRKCB
ANXA7
SOS1
NGF
SHC1
PRKCA
FGFR2
MKI67
MGMT
BAG3
F
YWHAZ
SPAG1
PRKCA
AKT1
PIK3CA
PIK3R1
PTK2
HRAS
GRB2
SOS1
NTRK1
PLCG1
NGF
SHC1
G
TERT
THEM4
DAB2
PIK3R1
PRKCA
GRB2
AKT1
PRKCB
CTSS
PLCG1
PIK3CA
NTRK1
HRAS
SOS1
SHC1
NGF
H
LIFR
IL7R
NGF
NTRK1
PRKCA
PLCG1
CISH
SOS1
SHC1
GRB2
AKT1
HRAS
PRKCD
PRKCB
PIK3R1
PIK3CA
RASSF1
DAB2
GHR
I
HGS
PDE6G
CD7
PLCG1
ITGB4
GRB2
PIK3R1
AKT1
GFAP
RPS6KB1
PRKCA
PIK3CA
NTRK1
HRAS
SOS1
SHC1
NGF
J
HMOX1
EP300
IL2RB
PIK3R1
NGF
PIK3CA
AKT1
PRKCB
HRAS
GRB2
SOS1
PRKCA
THOC5
PLCG1
NTRK1
SHC1
GRAP2
CSF2RB
EWSR1
K
ADRB2
PDGFRB
GRB2
PLCG1
PIK3R1
PAM
PRKCA
PPARGC1B
AKT1
PIK3CA
NTRK1
HRAS
NGF
CDC25C
SOS1
SHC1
CSF1R
YAP1
L
GRM5
GRIA4
GRB2
CBL
PRKCA
PRKCB
HRAS
PLCG1
PIK3CA
SOS1
NGF
NTRK1
AKT1
PIK3R1
SHC1
MMP7
CD3E

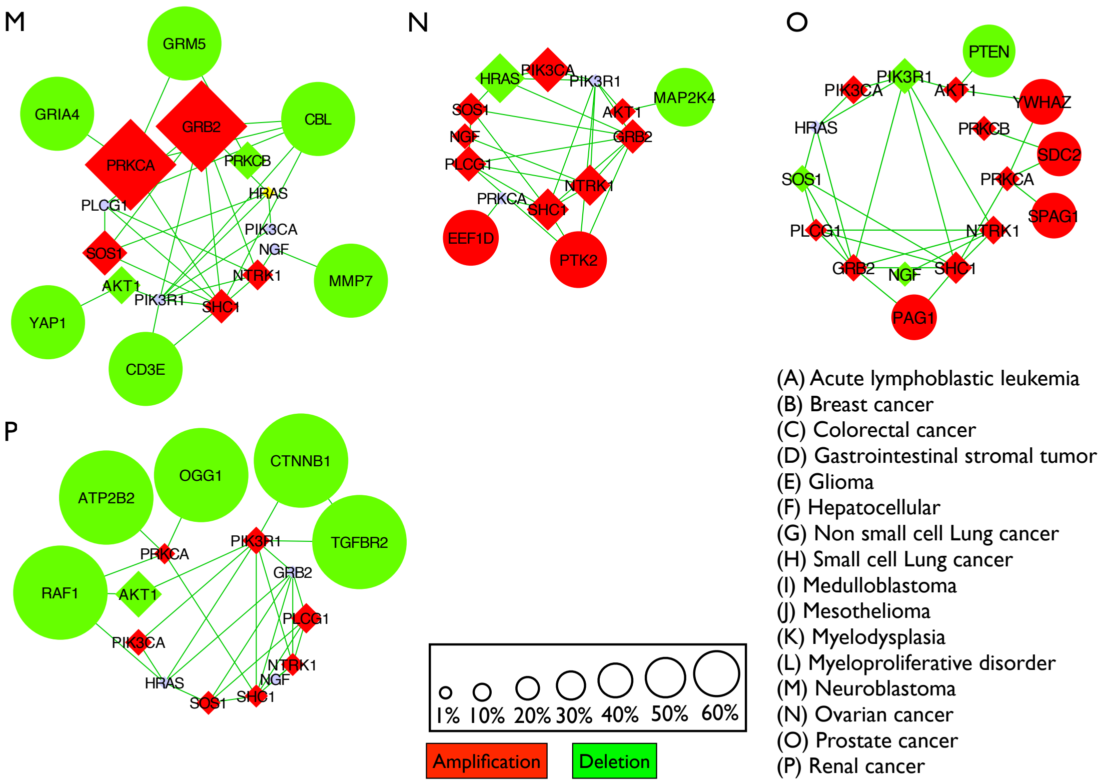

Supplementary Figure 5. NTRK1 (TrkA) signaling pathway is commonly disrupted by genes in copy number alterations across multiple types of cancers. This figure describes commonly disrupted TrkA signaling pathway across many cancer types. Red, and green colors indicate that genes are amplified, or deleted in copy number alterations, respectively. Gray color indicates that the gene is not altered by copy numbers. Diamond shape node represents that member genes in the pathway, and circular shape node represents genes that are interacting with member genes in the pathway in protein-protein interaction network. Size of nodes represents recurrent frequency of genes in copy number data (i.e. larger size of gene node indicates the gene is frequently altered in patient samples).

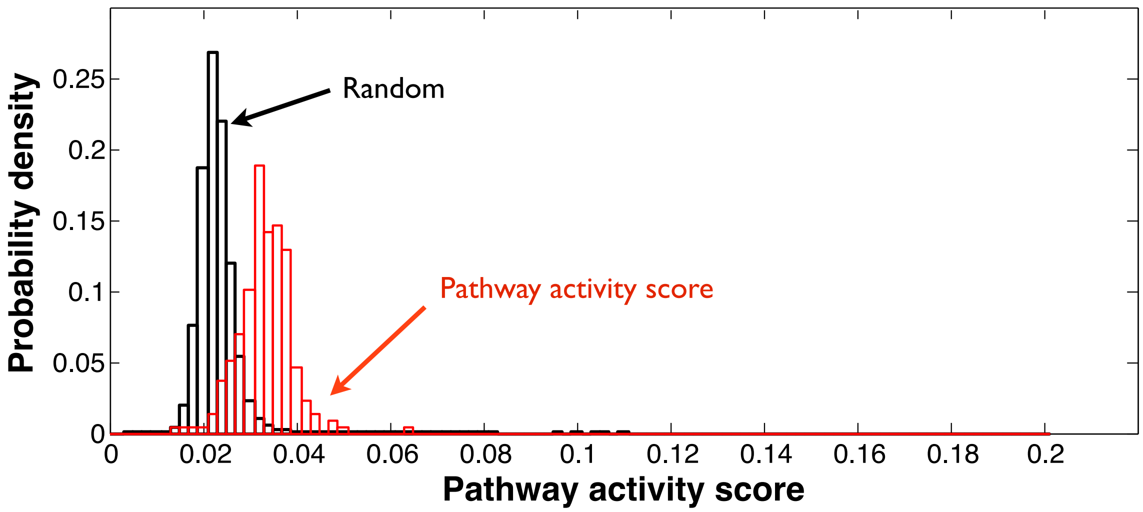

Supplementary Figure 6. Distribution of pathway activity scores. This figure describes a distribution of activity scores of pathways with a distribution of activity scores of pathways using randomization from Biocarta pathway database. A background distribution of randomization (black) is compared to a distribution of true pathway activity score (red). Pathways which have significant scores of true pathway activity score (on the right(red)) include TGF-beta, telomerase, NTRK1 and EGFR signaling pathways

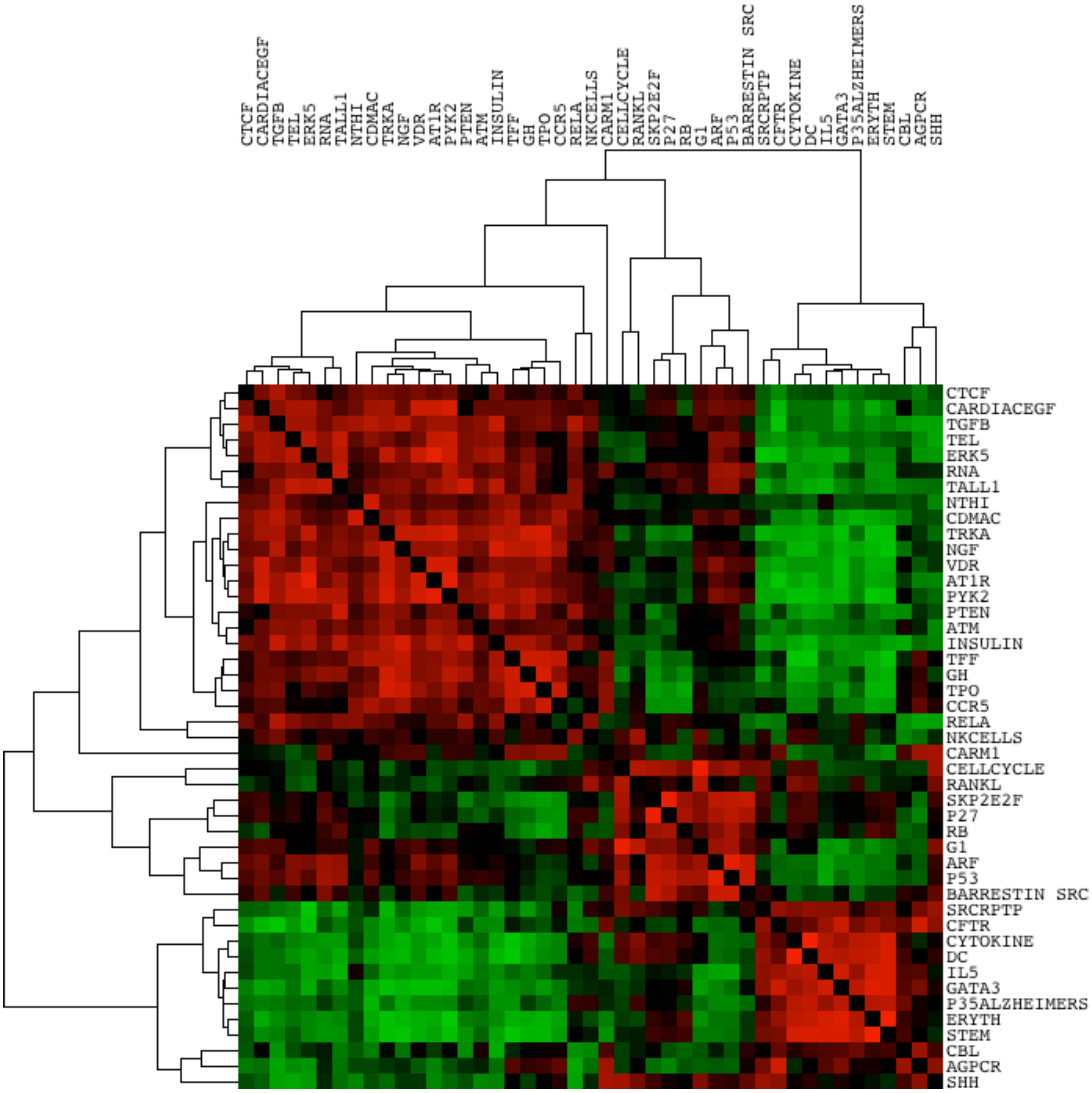

Supplementary Figure 7. Heat map describing the correlation coefficient of pathway co-disruption. This figure describes a distribution of activity scores of pathways with a distribution (red: positive correlation, green negative correlation). We collected top 3% (7 out of 217) disrupted pathways from the Biocarta pathway database in each cancer type, and found 45 disrupted pathways. We computed correlation coefficient of 47 pathways based on inferred pathway activity scores, and applied two-way hierarchical clustering using Cluster 3 with complete linkage to find co-disrupted pathway clusters.

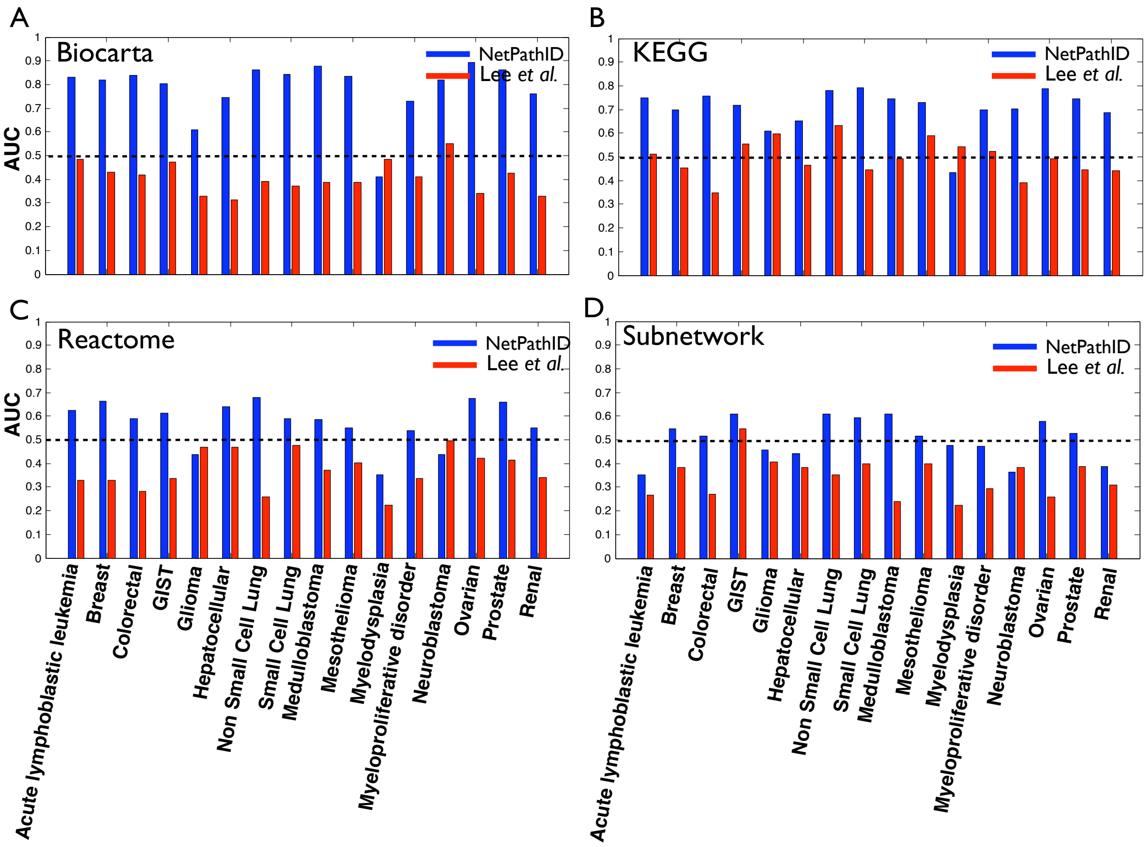

Supplementary Figure 8. False discovery rate using decoy pathways. Decoy pathways were created by assigning a new gene name to each gene in a pathway. NetPathID and Lee Lee *et al*. methods were then used to compute the activity of every pathway. We computed the areas under the curve (AUCs) based on rankings of the pathways for the methods in each pathway database. Since we are most interested in whether the true biological pathway is near the top, we report the AUC up to the first 50 false positives. Specifically, we calculated the percentage of the area under the ROC curve at 50 false positives. Each bar plot in each figure shows the AUC 50 for distinguishing real from decoy pathways using the perturbation ranking. For example, in breast cancer using Biocarta pathway database, AUC 50s are 0.821 and 0.452 for NetPathID and Lee et al method, respectively.

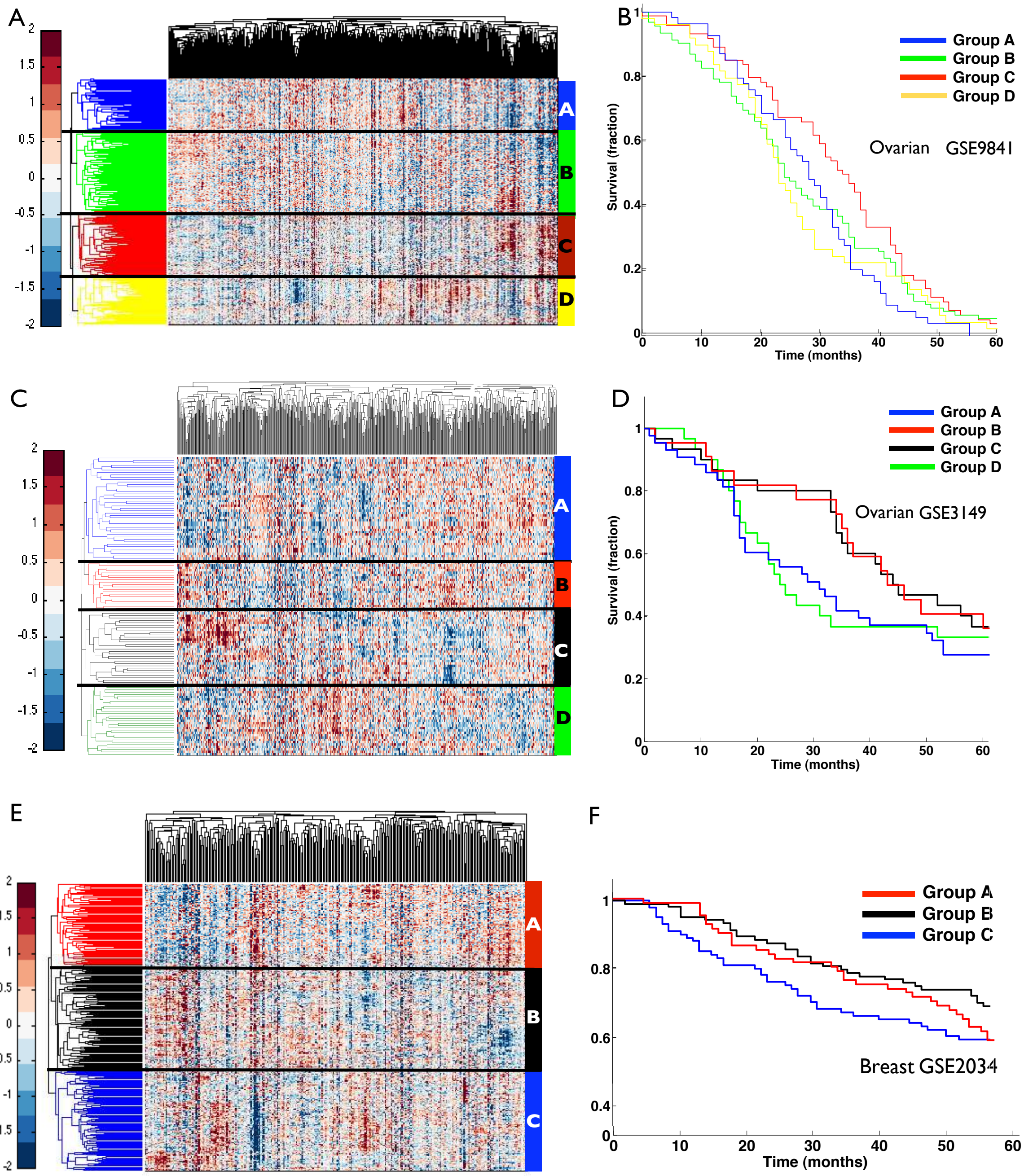

Supplementary Figure 9. Clustering and Kaplan-Meier analysis This figure describes two-way hierarchical clustering of two ovarian cancer datasets and breast cancer dataset using member genes in commonly disrupted pathways Kaplan-Meier survival plots for the clusters of patient subgroups from cancer microarray gene expression datasets (left) and colors indicate patient subgroups used for Kaplan-Meier analysis (right). (A, B): We found four patient subtypes, and Group C patients (median survival 35 months) have longer survival time, compared to Group A,

B, and D patients (median survival 29.5, 24, and 24 months, respectively). For instance, Group B and C patients show significantly different survival time (p-value < 0.01984, Hazard ratio = 1.3089). (C,D): While Group A (median survival: 18 months) and D (median survival: 19 months) have short survival time. Specifically, Group C and D show significantly different survival time (Hazard ratio: 1.4222, p-value <0.00906). (E,F): We found three patient subgroups in breast cancer dataset. Group C patients have short survival time, compared to Group A, and B patients (p-value <0.00475, Hazard ratio = 1.4847, median survival of Group A and C: 36 vs. 24 months).

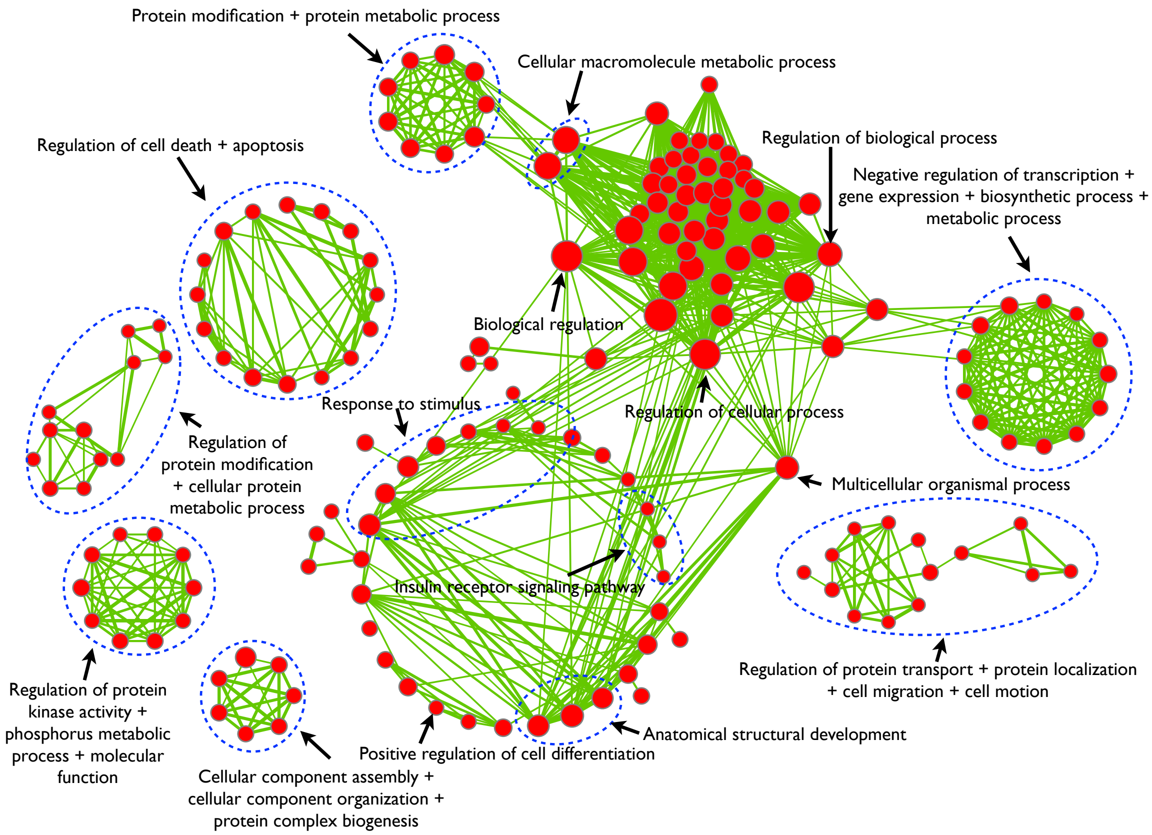

Supplementary Figure 10. A functional map of commonly disrupted pathways across cancers

Functional enrichment results of commonly disrupted pathways across cancers were mapped as a network of gene sets (nodes) related by mutual overlap (edges), where the color (red) indicates significance (FDR q-value) of gene sets. Node size is proportional to the total number of genes in each set and edge thickness represents the number of overlapping genes between sets. Groups of functionally related gene sets are circled and labeled with enriched GO biological processes manually. Enriched gene sets are coherent (e.g. enriched gene sets have similar biological processes and functions) and showed many cancer-related biological processes including regulation of cell death, apoptosis, cell migration, and cell differentiation.

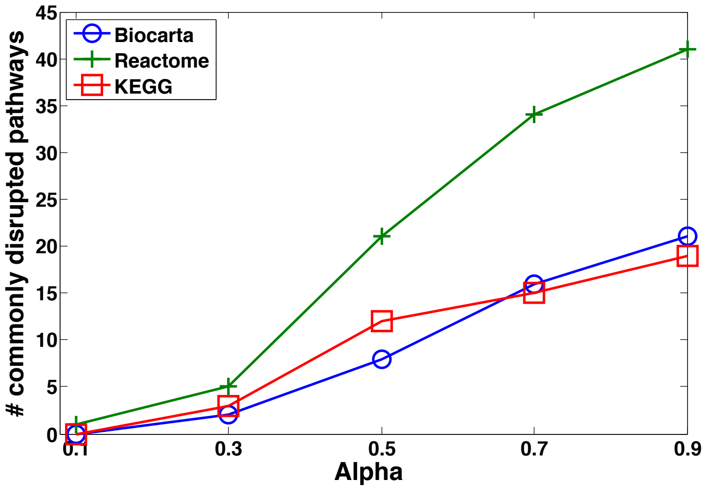

Supplementary Figure 11. Effects on different parameter choices of alpha. We tested five values for hyper parameter alpha to the label propagation. One interesting observation is that the alpha parameter influences the number of commonly disrupted pathways: the larger alpha parameter, more commonly disrupted pathways. This can be explained by the optimization formulation in equation (1); when the alpha is large, we put more weights on the cluster structures in the protein-protein interaction network, and thus label propagation favors the modularity structure in the protein-protein interaction network by assigning highly consistent activity scores to the densely connected genes. In other words, those genes that are densely connected in the gene modules (e.g. member genes in the pathways) will get highly consistent activity scores. In contrast, when the alpha is close to 0, the label propagation is similar to simply overlaying activity score to genes without exploring the cluster structures in the protein-protein interaction networks.
