# Supplementary material for: Large-scale integrative network-based analysis identifies common pathways disrupted by copy number alterations across cancers
Source: BMC Genomics. 2013 Jul 3;14:440. doi: 10.1186/1471-2164-14-440 (PMC3703268; doi:10.1186/1471-2164-14-440)
Supplement: Additional file 2 — Comparisons with Lee et al. method and overrepresentation-based enrichment methods, and HotNet. [file 1471-2164-14-440-S2.doc]

# [Supplementary Information] Large-scale integrative network-based analysis identifies common pathways disrupted by copy number alterations across cancers

### TaeHyun Hwang^†1^, Gowtham Atluri^2^, Rui Kuang^2^, Vipin Kumar^2^, Timothy Starr^1,4^, Kevin A. T. Silverstein^1^, Peter M Haverty^3^, Zemin Zhang^3^, Jinfeng Liu^†^*^3^*

^1^Masonic Cancer Center, University of Minnesota – Twin Cities, Minneapolis, Minnesota, Unites States of America

^2^Department of Computer Science and Engineering, University of Minnesota – Twin Cities, Minneapolis, Minnesota, Unites States of America

^3^Department of Bioinformatics and Computational Biology, Genentech Inc., South San Francisco, California, Unites States of America

^4^Department of Obstetrics, Gynecology & Women's Health, University of Minnesota, Minneapolis, Minnesota, United States of America

^†^Corresponding author

Email addresses:

THH: [hwang071@umn.edu](mailto:charles@darwin.co.uk)

GA: [gowtham@cs.umn.edu](mailto:gowtham@cs.umn.edu)

RK: [kuang@cs.umn.edu](mailto:kuang@cs.umn.edu)

VK: [kumar@cs.umn.edu](mailto:kumar@cs.umn.edu)

TS: [star0044@umn.edu](mailto:star0044@umn.edu)

KS: [silve023@umn.edu](mailto:silve023@umn.edu)

PMH: [phaverty@gene.com](mailto:phaverty@gene.com)

ZZ: [zhang.zemin@gene.com](mailto:zhang.zemin@gene.com)

JL: [liu.jinfeng@gene.com](mailto:liu.jinfeng@gene.com)

### Commonly disrupted pathways are related to cancer biology

1. We examined commonly disrupted pathways identified by NetPathID in terms of their functional coverage and relevance. The functional enrichment of the genes in the commonly disrupted pathways was measured based on significant overlap with biological processes annotated in Gene Ontology using the DAVID tool (see Supplementary Table 5~12 for functional enrichment results). Supplementary Fig. 10 shows a network view of GO biological functions enriched with 330 genes in 42 commonly disrupted pathways derived from subnetwork modules (FDR < 0.001). Each node in Fig. 10 refers to a gene set containing genes that share a common biological process or operate in the same pathway. Many GO biological process terms known to be involved in cancer development and progression including cell death, differentiation, migration, apoptosis, and growth factor signaling are significantly enriched. These results suggest that despite the heterogeneity of cancers in clinical behavior or genomic profiles, many cancer types share a set of common biological processes for cancer development.

# Comparison to Lee et al’s method and overrepresentation-based enrichment methods

1. To evaluate the performance of NetPathID to discover disrupted pathways, we compared NetPathID with two other methods, which we refer to as baselines. The first method is conventional overrepresentation-based enrichment using hypergeometric testing with copy number data. For the second baseline, we used a pathway-based method developed by Lee E. and colleagues , since this method is directly applicable in our context, and comparable to NetPathID. We adopted and implemented the method in to discover disrupted pathways across cancers. To perform overrepresentation-based enrichment analysis, we collected the top 1% ranked genes (189 out of 18932 genes) based on their –log10(qval) using GISTIC in each type of cancer. We then reported significantly enriched pathways with top ranked genes using hypergeometric testing.
2. We first investigated whether the methods can accurately identify cancer-related pathways from negative controls. Specifically, to estimate the false discovery rate of discovering cancer-related pathways, we used simulated ‘decoy’ pathways as negative controls as suggested by . We replaced member genes in pathways by randomly selecting the same number of genes in the genome, and constructed a set of decoy pathways. Then, we applied NetPathID and Lee et al. methods to compute activity scores of both real and decoy pathways, and ranked them by computed activity scores. We found that NetPathID ranked many more real pathways higher than decoy pathways, compared to the Lee *et al.* method (Supplementary Figure 8). For example, among the top 50 Biocarta pathways by NetPathID, only two are decoy pathways, whereas 32 out of the top 50 pathways by Lee et al method are decoy. Similar results were obtained from KEGG and Reactome pathways as well. We also measured the performance by calculating the areas under the curve (AUC) of receiver operating characteristics. The comparison of AUC across cancers from Biocarta, KEGG, and Reactome pathway databases showed that NetPathID consistently outperformed Lee *et al.* method in all experiments (Supplementary Figure 8).
3. To provide a comparison in terms of the discovery of commonly disrupted pathways, we applied the same criteria to overrepresentation-based enrichment analysis and Lee *et al*. methods to report commonly disrupted pathways across cancers. We also performed aggregated and pooled analysis suggested by to report disrupted pathways enriched based on commonly altered genes across cancers (see Methods). Most of the commonly disrupted pathways identified from NetPathID are related to cancer biology (Supplementary Table 1). In contrast, the top disrupted pathways from either aggregated or pooled analysis do not appear to be cancer-related (Supplementary Table 2). This implies that simply overlaying pathway membership information with copy number changes without considering interactions or dependencies among genes would fail to discover biologically relevant cancer-related pathways. The method proposed by Lee et al. only identified an average of one commonly disrupted pathway, compared to 14 commonly disrupted pathways identified by NetPathID (Supplementary Table 3). Overrepresentation-based enrichment analysis could not identify any commonly disrupted pathways.

# HotNet

1. HotNet  is aimed at discovering significant subnetworks from a gene interaction network based on somatic mutation rates in a patient population. This approach is used to discover novel gene subnetworks whose disruption potentially leads to cancer. We applied HotNet to discover novel gene subnetworks that have surprisingly more mutations than by random chance in several type of cancers. HotNet has two key parameters: i) t, for computing an influence matrix from the PPI network, ii) delta for thresholding the influence matrix to drop weak edges. We used multiple combinations of t and delta to discover disrupted subnetworks: i) t=200 and delta = 0.1, ii) t=200 and delta = 0.05, iii) t=50 and delta = 0.5. We found that in all of these combinations, for each cancer type most of the subnetworks discovered have less than four member genes. Moreover, these subnetworks have very high probabilities (>0.5), suggesting that these disrupted subnetworks can be discovered by random chance. This indicates that HotNet would not be applicable for our analysis. Note that we provide all results from HotNet in our supplementary website (<http://compbio.cs.umn.edu/NetPathID/>).

### Cancer-related gene enrichment analysis with baselines

We investigated the commonly disrupted pathways in terms of their enrichment for known cancer-related genes with two baselines (Lee et al’s method, and HotNet). We found that the cancer gene fraction of the functional modules from the two baselines is even smaller than in the random cases..

1. One possible reason for the poor performance of Lee et al. method ([Lee, Chuang et al. 2008](#_ENREF_23)) using gene scores from aggregated and pooled analysis is that the method is not designed to identify pathways using copy number alteration. Instead, it was designed for gene expression data. Thus, a simple adaptation of Lee et al. method ([Lee, Chuang et al. 2008](#_ENREF_23)) to analyze copy number alteration data may not be appropriate. We also applied the HotNet method , which integrates copy number alterations with protein-protein interaction networks to identify significantly mutated subnetworks. However, most of identified subnetworks contain less than five member genes, and are not statistically significant. One potential reason for the poor performance of HotNet is that most of the frequently altered genes were spread across the network and were not connected directly or by relatively short paths (given the small world property of protein interaction networks). This makes it difficult for the HotNet ([Vandin, Upfal et al. 2010](#_ENREF_46)) to identify significantly mutated subnetworks containing many altered genes

### Bias in network

To test the robustness of NetPathID with regards to the bias in the protein-protein interaction networks, we repeated the experiments on extended protein-protein interaction networks. It is known that well-studied disease proteins tend to have more interactions in the protein-protein interaction networks, and this degree bias could potentially lead to superior performance of the network-based methods. An extended protein-protein interaction network with the same degree of interactions for each protein was generated to assess the influence of the bias as suggested by . The extended protein-protein interaction network was constructed by combining the HPRD, OPHID, BIND, and MINT databases containing 72,431 undirected binary interactions between 14,433 human proteins . We applied NetPathID to the extended protein-protein interaction network to assess whether NetPathID relies on the degree of interactions of genes in the network to identify commonly disrupted pathways. We report results using the extended protein-protein interaction networks in Supplementary Table 4. Interestingly, NetPathID could discover many commonly disrupted pathways that are identified from the initial analysis using the original protein-protein interaction networks. This indicates that NetPathID does not simply rely on the bias of degree of interactions of genes in the protein-protein interaction network to discover commonly disrupted pathways.

### GISTIC with different cutoffs

To test the sensitiveness of NetPathID with regards to the different cutoffs with GISTIC (e.g., amplication > 0.1, 0.3, or 0.5, and deletion <-0.1, -0.3, or -0.5), we repeated experiments using different GISTIC cutoffs, while other parameters are not changed. Interestingly, the rankings of commonly disrupted pathways using GISTIC cutoffs amplication > 0.3 & deletion <-0.3 and amplciation > 0.5 & deletion < -0.5 are very consistent with results with GISTIC default cutoff amplication > 0.1 & deletion < -0.1. For example, telomerase, NTRK1 (TRKA) and TGF-beta signaling pathways from Biocrata pathway are consistently top ranked across more than 10 types of cancers from experiments with GISTIC cutoff amplication > 0.3 & deletion < -0.3, and many other commonly disrupted pathways identified by the analysis with GISTIC default cutoff are also consistently found across experiments with various GITIC cutoffs (see Supplementary Table 15). We also found that that the ranking of disrupted pathways in each type of cancers are stable across different GISTIC cutoffs. For example, at least 11 cancer types show that more than 50% of disrupted pathways from Biocarta, KEGG, Reactome, and PPI subnetworks in each cancer type and are consistently ranked within the top 20% across all experiments with different GISTIC cutoffs (see Supplementary Table 17). More specifically, an average 64.7% of disrupted pathways from Biocarta, KEGG, Reactome, and PPI subnetworks in each cancer type are consistently ranked within top 20% on experiments using GISTIC cutoff amplification > 0.3 & deletion < -0.3. And an average 58.8% of disrupted pathways from Biocarta, KEGG, Reactome, and PPI subnetworks in each cancer type are consistently ranked within top 20% on experiments using GISTIC cutoff amplification > 0.5 & deletion < -0.5. These observations suggest that our method utilizing two filtering steps (e.g., a set of seed genes detected by GISTIC and the average log ratio of the amplified or deleted gene) is less sensitive to input data from GISTIC with different cutoffs. Moreover, our method can use a set of seed genes detected by other tools to identify significantly altered genes based on copy number changes. The code to generate results using GISTIC different cutoffs is available at http://compbio.cs.umn.edu/NetPathID/code.zip. We also provide results from GISTIC with different cutoffs at http://compbio.cs.umn.edu/NetPathID/GISTIC_cutoffs.zip.

### Removing arm-level copy number alterations

Arm-level copy number alterations are often observed in large-scale copy number analyses, and show very strong evidence of selective gain or loss in the specific cancer types . In this study, we also found that some cancers show arm-level copy number alterations. To test whether or not whole arm chromosome loss or gain significantly affects the list of pathways identified by NetPathID we repeated our analysis without including whole arm chromosome losses or gains. Interestingly, we observed that most of the commonly disrupted pathways are identified even when we exclude arm-level copy number alterations, while the frequency of commonly disrupted pathways varied moderately (see Supplementary Table 19). We also found that the lists of top ranked disrupted pathways in each type of cancer between the two experiments are very consistent. For example, most cancer types show that more than 70% of disrupted pathways from Biocarta, KEGG, Reactome, and PPI subnetworks in each cancer type are consistently ranked within top 20% in both experiments (see Supplementary Table 20 and 21). This suggests that while some cancer types may present arm-level copy number alterations, there is enough genetic information in the remaining copy number data to successfully identify the pathways driving that cancer.

# References

1. Lee, E., H. Y. Chuang, et al. (2008). "Inferring pathway activity toward precise disease classification." PLoS Computational Biology **4**(11): e1000217.
2. Beroukhim, R., G. Getz, et al. (2007). "Assessing the significance of chromosomal aberrations in cancer: methodology and application to glioma." Proceedings of the National Academy of Sciences **104**(50): 20007.
3. Vaske, C. J., S. C. Benz, et al. (2010). "Inference of patient-specific pathway activities from multi-dimensional cancer genomics data using PARADIGM." Bioinformatics **26**(12): i237.
4. Beroukhim, R., C. H. Mermel, et al. (2010). "The landscape of somatic copy-number alteration across human cancers." Nature **463**(7283): 899.
5. Vandin, F., E. Upfal, et al. (2010). Algorithms for detecting significantly mutated pathways in cancer, Springer.
6. Wu, X., R. Jiang, et al. (2008). "Network-based global inference of human disease genes." Molecular systems biology **4**(1).
